# Cardiac telocytes inhibit cardiac microvascular endothelial cell apoptosis through exosomal miRNA-21-5p-targeted *cdip1* silencing to improve angiogenesis following myocardial infarction

**DOI:** 10.7150/thno.47021

**Published:** 2021-01-01

**Authors:** Zhaofu Liao, Yilin Chen, Chuncui Duan, Kuikui Zhu, Ruijin Huang, Hui Zhao, Maik Hintze, Qin Pu, Ziqiang Yuan, Luocheng Lv, Hongyi Chen, Binglin Lai, Shanshan Feng, Xufeng Qi, Dongqing Cai

**Affiliations:** 1 The First Affiliated Hospital, Key Laboratory of Regenerative Medicine, Ministry of Education, Jinan University, Guangzhou 510632, China.; 2 Key Laboratory of Regenerative Medicine, Ministry of Education, Jinan University, Guangzhou 510632, China.; 3 Joint Laboratory for Regenerative Medicine, Chinese University of Hong Kong-Jinan University, Guangzhou 510632, China.; 4 International Base of Collaboration for Science and Technology (JNU), Ministry of Science and Technology, Guangdong Province, Guangzhou 510632, China.; 5 Department of Developmental and Regenerative Biology, Jinan University, Guangzhou 510632, China.; 6 Institute of Anatomy, Department of Neuroanatomy, University of Bonn, Germany.; 7 Stem Cell and Regeneration TRP, School of Biomedical Sciences, Chinese University of Hong Kong, Hong Kong.; 8 Medical Department, MSH Medical School Hamburg, Germany.; 9 Cancer Institute of New Jersey, Department of Medical Oncology, Robert Wood Johnson of Medical School, USA.

**Keywords:** cardiac telocytes, apoptosis of endothelial cells, regeneration of myocardial infarction, exosomal miR-21, Cdip1 gene

## Abstract

Promotion of cardiac angiogenesis in ischemic myocardium is a critical strategy for repairing and regenerating the myocardium after myocardial infarction (MI). Currently, effective methods to aid in the survival of endothelial cells, to avoid apoptosis in ischemic myocardium and to achieve long-term cardiac angiogenesis are still being pursued. Here, we investigated whether cardiac telocyte (CT)-endothelial cell communication suppresses apoptosis and promotes the survival of endothelial cells to facilitate cardiac angiogenesis during MI.

**Methods:** CT exosomes were isolated from CT conditioned medium, and their miRNA profile was characterized by small RNA sequencing. A rat model of left anterior descending coronary artery ligation (LAD)-mediated MI was assessed with histology for infarct size and fibrosis, immunostaining for angiogenesis and cell apoptosis and echocardiography to evaluate the therapeutic effects. Cardiac microvascular endothelial cells (CMECs) and the LAD-MI model treated with CT exosomes or CT exosomal miRNA-21-5p *in vitro* and *in vivo* were assessed with cellular and molecular techniques to demonstrate the underlying mechanism.

**Results:** CTs exert therapeutic effects on MI *via* the potent paracrine effects of CT exosomes to facilitate the inhibition of apoptosis and survival of CMECs and promote cardiac angiogenesis. A novel mechanism of CTs is revealed, in which CT-endothelial cell communication suppresses apoptosis and promotes the survival of endothelial cells in the pathophysiological myocardium. CT exosomal miRNA-21-5p targeted and silenced the cell death inducing p53 target 1 (*Cdip1*) gene and thus down-regulated the activated caspase-3, which then inhibited the apoptosis of recipient endothelial cells under ischemic and hypoxic conditions, facilitating angiogenesis and regeneration following MI.

**Conclusions:** The present study is the first to show that CTs inhibit cardiac microvascular endothelial cell apoptosis through exosomal miRNA-21-5p-targeted *Cdip1* silencing to improve angiogenesis in myocardial infarction. It is believed that these novel findings and the discovery of cellular and molecular mechanisms will provide new opportunities to tailor novel cardiac cell therapies and cell-free therapies for the functional and structural regeneration of the injured myocardium.

## Introduction

Regeneration of myocardial infarction (MI), a major cause of morbidity and mortality worldwide, remains a substantial clinical challenge. Effective strategies to promote survival and avoid apoptosis of endothelial cells in the ischemic myocardium could help to achieve long-term cardiac angiogenesis. In the hostile ischemic microenvironment during MI, most cardiac cells, including cardiomyocytes, endothelial cells and macrophages, were shown to undergo apoptosis 1, 2. In a nonhuman primate model of MI and heart failure (HF), except for cardiomyocyte apoptosis, nonmyocyte apoptosis was shown to account for the majority of apoptotic cells in both adjacent and remote areas, while macrophages comprised the largest fraction of apoptotic nonmyocytes (41% vs. 18% neutrophils, 25% endothelial and other cells, and 16% fibroblast-like cells) 1. Indeed, the unexpectedly high level of nonmyocyte apoptosis was confirmed in myocardial biopsies from patients with congestive cardiomyopathy and HF 1, while apoptotic endothelial cells, interstitial cells and fibroblast-like cells, but rarely apoptotic myocytes, were detected in the right ventricle of a murine model of right ventricular volume overload 3. These findings suggest that endothelial cells, such as cardiac microvascular endothelial cells (CMECs), comprise up to one-third of the total heart cells and play a critical role in maintaining and supporting coronary microvessels and adjacent cardiomyocytes under normal conditions and angiogenesis under pathophysiological conditions. Apoptosis induced by ischemic injury is an important pathophysiological event in MI and MI regeneration. Indeed, it has been revealed that endothelial cells are more prone to incur damage than cardiomyocytes during ischemia and reperfusion injury 4-6, while apoptosis of CMECs as well as other endothelial cell beds induced by ischemia and hypoxia injury precedes cardiomyocyte apoptosis and the release of soluble proapoptotic mediators from endothelial cells in an ischemic environment and can promote cardiomyocyte apoptosis 7. These findings suggest that protecting endothelial cells, especially CMECs, from apoptosis under MI will be one method of promoting cardiac angiogenesis and achieving more effective regeneration of MI. Accordingly, effective strategies for repressing CMEC apoptosis have recently been pursued to develop more effective means to regenerate MI and prevent post-MI heart failure.

Recent progress in the field has revealed that cells (cardiomyocytes and noncardiomyocytes, such as endothelial cells and other interstitial cell types) within the supporting niche of the myocardium play an important role in supporting cardiac physiopathology and myocardial regeneration 8. A novel population of cardiac interstitial cells named telocytes has recently been identified in the heart interstitium 9-18. Recently, we demonstrated that following permanent coronary occlusion, a significant number of CTs in the infarct zone undergo cell death, and the CT network in the ischemic area is destroyed. The therapeutic transplantation of CTs in rats with acute MI has been shown to decrease infarct size and improve myocardial function in the short term (2 weeks) and mid-term (14 weeks) by promoting cardiac angiogenesis and reconstruction of the CT network and decreasing cardiac fibrosis 19, 20. Accordingly, in this study, we further hypothesize that CTs might play an important role in the endogenous angiogenic potential in the myocardium, and one mechanism underlying CT-mediated cardiac angiogenesis for MI after CT transplantation might be CT-derived exosomes (CT-exos), which repress apoptosis *via* CT-CMEC communication. Indeed, we report here for the first time that CTs induce antiapoptotic effects for CMECs *via* CT exosomal miRNA-21-5p-targeted *Cdip1* silencing to inhibit caspase-3 activation and thus improve angiogenesis and regeneration following MI.

## Methods

### Animals

Three-month-old female Sprague-Dawley (SD) rats (250-300 g) were utilized in the present study. The rats were housed for 2 weeks to allow them to adapt before experimentation. They were provided with food and water *ad libitum*. Animal care, surgery and handling procedures were performed according to regulations established by The Ministry of Science and Technology of the People's Republic of China ([2006] 398) and approved by Jinan University Animal Care Committee.

### Isolation and phenotypic confirmation of cardiac telocytes

Young (3-month-old) female SD rats were used to isolate CTs using a previously described method 19, 20. Briefly, the hearts were isolated and minced, and then, the tissues were treated with 2.5 mL of DMEM supplemented with 0.05% collagenase P (cat. no. 11213865001; Roche) and 0.1% trypsin (cat. no. 0458; Amresco) and incubated for 10 min at 37 °C on a shaker (180 rpm). After the supernatant was removed, collagenase and trypsin medium were added, and the mixture was incubated at 37 °C on a shaker for 45 min. The digested tissue was dissociated by gentle pipetting every 15 min. The supernatant was then sequentially filtered through a 100-µm and a 41-µm nylon mesh, and the collected cell suspension was centrifuged at 50 × *g* for 2 min. The supernatant was then removed and recentrifuged at 300 × *g* for 10 min. The pellet was resuspended in 5 mL of PEB medium (PBS supplemented with 0.5% bovine serum albumin and 2 mM EDTA [pH 7.2]). The mixture was then centrifuged at 38 × *g* for 2 min to remove the debris, and the collected supernatant was further centrifuged at 200 × *g* for 10 min. The cell pellet was then mixed with 1 mL of PEB and 5 µL of a rabbit anti-rat C-kit antibody (1:50; cat. no. PA5-16770; Thermo Fisher), and the sample was incubated at 4 °C for 40 min. An additional 2 mL of PEB was then added, and the mixture was centrifuged at 458 × *g* for 4 min to collect the cells. The pellet was resuspended in 160 µL of PEB, and 20 µL of a solution containing goat anti-rabbit IgG microbeads (cat. no. 5111007039; Miltenyi Biotec) was added, followed by incubation at 4 °C for 25 min. The mixture was then added to a magnetic separation (MS) column (Miltenyi Biotec) in a magnetic field, and the unlabeled cells were allowed to pass through. The MS column was then removed from the magnetic field, and the labeled cells were flushed out with PEB. The isolated cell pellet was collected by centrifugation at 458 × *g* for 4 min, followed by culture in DMEM containing 20% fetal calf serum at 37 °C and 5% CO_2_ in a 95% air incubator. With this method, more than 93% of the isolated cells were C-kit^+^ and CD34^+^ 19, 20. In this study, only isolated CTs at passage 5 or less were used for the experiments.

For phenotypic confirmation, the isolated cells collected by the above protocol were seeded in lysine-treated coverslips and cultured with DMEM containing 20% fetal calf serum at 37 °C and 5% CO_2_ in a 95% air incubator. As the unique morphology is a critical standard for identification of the CTs, the cultured cells were assessed by microscopy in the earliest passage (Passage-0) 2 days after seeding. It was found that most of the cells had piriform/spindle/triangular cell bodies containing long and slender telopods with the alternation of thick segments (podoms) and thin segments (podomers) (Figure S1A). At present, there is no unique marker for distinguishing CTs by the expression of a single protein, and the most commonly used markers are c-kit, CD34, vimentin and PDGFRA 21-26. Therefore, double immunofluorescence staining, which was conducted with anti-c-Kit+anti-CD34 (1:50; cat. no. PA5-16770; Thermo Fisher vs. 1:50; cat. no. AF4117; R&D) and anti-c-Kit+anti-vimentin (1:50; cat. no. PA5-16770; Thermo Fisher vs. 1:100; cat. no. ab8978; Abcam) for the collected Passage-0 cells was applied. The collected cells were positive for c-Kit, CD34 and vimentin (low expression) (Figure S1B a1-4 and Figure S1C b1-4) and showed commonly used telocyte markers. As it was reported that c-Kit^+^ and/or CD34^+^ endothelial cells were found in the myocardium 18, 27, to exclude the possibility that the c-Kit^+^ endothelial cells were mixed with the collected CTs during sorting, we performed double immunofluorescence staining with different combinations (anti-c-Kit+anti-CD31 [1:50; cat. no. sc-365504; Santa Cruz vs. 1:100; cat. no. 11265-1; Proteintech], anti-c-Kit+vWF [1:50; cat. no. sc-365504; Santa Cruz vs. 1:100; cat. no. F3520; Sigma]) for the collected Passage-0 cells. The collected cells were negative for CD31 and vWF, well-established markers of endothelial cells (Figure S1B c1-4 and d1-4). Considering that c-Kit and CD34 are also recognized as markers for hematopoietic lineage stem cells or progenitor stem cells 21, we observed the potential characteristics of stem cells in the collected CTs by colony formation and successive passage assays for proliferation potential as well as mesoderm differentiation assays. The results indicated that the potency of colony formation and proliferation of the collected CTs did not meet the standards for stem cells, as we found that the cultured CTs did not show colony formation (Figure S1C) and entered growth arrest in approximately 10-15 passages when they were serially subcultured (data not shown). Furthermore, the results of the differentiation assay showed that the collected cells (passages 1~2) failed to be induced to differentiate into mesoderm-derived cells, such as bone and fat cells (Figure S1D). The bone cell differentiation protocol was as follows: DMEM included 10% FBS, dexamethasone (1 µM), ascorbic acid (50 µg/mL), β-glycerophosphate (10 mM), and 1-α,25-dihydroxyvitamin D (10 nM), the cells were treated for 16 days at 37 °C and 5% CO_2_ in a 95% air incubator, and osteogenic differentiation was identified by Alizarin red S staining. The fat cell differentiation protocol was as follows: DMEM included 10% FBS, dexamethasone (1 μM), 3-isobutyl-1-methylxanthine (0.5 mM) and insulin (5 µg/mL), the cells were treated for 16 days at 37 °C and 5% CO_2_ in a 95% air incubator, and fat cell differentiation was identified by oil red O staining. In addition, the individual isolated cells, which possess the hallmark morphology of telocytes in its Passage-0 stage, as shown in Figure S1A, were collected using a micromanipulator under microscopy. Single-cell RNA sequencing was applied for gene expression profile analysis (detailed method not shown here). Our preliminary results for 11 collected single cells revealed that all 11 cells were negative for the common stem cell markers Oct-4, SSEA-1 and Sca-1. In addition, the cells were positive for CD105, CD73 and Col1a1 (Figure S1E), which are commonly expressed by mesenchymal linage-derived interstitial cells. The positive expression of single-cell sequencing was set at ≥ 10 FPKM. All of the above evidence strongly indicates that the CTs applied in this study are consistent with the commonly accepted morphology and molecular marker features of telocytes 21-26. These cells are more similar to interstitial cells than endothelial cells and progenitor stem cells, as they are negative for CD31 and vWF, and the progenitor or stem cell characteristics are not notable, although the cells were positive for c-Kit and CD34.

### Isolation of CMECs from adult rat hearts

Young (3-month-old) female Sprague-Dawley rats were used. The hearts were removed, and CMECs were isolated and cultured in CMEC medium (medium 199 basic supplemented with 10% fetal bovine serum, 100 U/mL penicillin, 100 μg/mL streptomycin and endothelial cell growth supplement) as described in our previous report 28, 29.

### Media conditioning and exosome purification

CT cells were grown in 100-mm dishes at 37 °C in 5% CO_2_ and 95% air_,_ in DMEM containing 1% penicillin, streptomycin and 20% FBS until they reached 80-90% confluence, and then, the culture medium was removed. After three washes with PBS (pH 7.4), DMEM containing 20% exosome-free serum was added, and the cells were incubated at 37 °C in 5% CO_2_ and 95% air for 48 h. Ten milliliters of conditioned medium was harvested from different dishes and centrifuged at 3000 × *g* for 15 min to pellet the cells before filtering through a 0.22-µm filter to remove cell debris. The collected medium was used to extract CT-derived exosomes (CT-exos) with ExoQuick-TC Exosome Precipitation Solution (cat. no. EXOTC50A-1; SBI) according to the manufacturer's instructions. Briefly, 2 mL of ExoQuick-TC Exosome Precipitation Solution was mixed with the collected medium, incubated at 4 °C overnight, and then centrifuged at 1500 × *g* for 30 min. After the supernatant was removed, the pellet was recentrifuged at 1500 × *g* for 5 min to remove the residual fluid, and then, the CT-exos were resuspended in PBS (pH 7.4).

For generation of miR-21-5p-deficient exosomes (CT-exos-miR21KO) or miR-21-5p-overexpressing exosomes (CT-exos-miR21OE), CTs were transfected in suspension with antagomir miR-21-5p inhibitor (5'-UCAACAUCAGUCUGAUAAGCUA-3'; 100 nM; GenePharma) or agomir miR-21-5p mimic (5'-UAGCUUAUCAGACUGAUGUUGA-3'; 100 nM; GenePharma) and seeded onto 100-mm dishes. Exosomes were isolated from DMEM containing 20% exosome-free serum conditioned media (48 h conditioning) as described in the protocol above.

### Transmission electron microscopy for CTs and CT exosomes

Tissue samples of the left ventricular myocardium of female SD rats were obtained and processed for transmission electron microscopy (TEM) to observe the unique morphology of CTs. Briefly, tissue specimens (approximately 1 mm^3^) were fixed with 4% glutaraldehyde (in 0.1 M cacodylate buffer, pH 7.3) for 24 h at 4 °C. After a brief wash in 0.1 M cacodylate buffer (CB), the tissue samples were postfixed with 1% osmium tetroxide in 0.1 M CB for 1 h at 4 °C, followed by dehydration in a graded series of ethanol solutions. After impregnation with propylene oxide, the samples were immersed overnight in a mixture of propylene oxide and Epon, followed by embedding in Epon. Ultrathin sections were cut and collected on formvar-coated copper grids, stained with uranyl acetate and lead citrate, and observed using TEM (Philips Tecnai 10 TEM).

Three microliters of isolated CT-exos was placed on formvar carbon-coated 200-mesh copper electron microscopy grids, incubated at room temperature for 5 min, and then subjected to standard uranyl acetate staining. The grid was washed with PBS three times and allowed to become semidry at room temperature before observation. The morphology and diameter of the CT-exos were analyzed using TEM (Philips Tecnai 10 TEM).

### Scanning electron microscopy for CT exosomes

The isolated CT-exos suspended in PBS were adhered to Formvar-coated copper grids. The preparations were attached to metal stubs and coated with gold to a thickness of 15 nm. The morphology and diameter of the CT-exos were analyzed using a Philips XL30 scanning electron microscope (SEM).

### Atomic force microscopy for CT exosomes

For atomic force microscopy (AFM) analysis, the isolated CT-exos were diluted 1:100 in deionized water and adsorbed to freshly cleaved mica sheets for 10 min. The sheets were rinsed thoroughly with deionized water to remove unbound CT-exos and dried under a gentle stream of nitrogen. Bioscope II (Veeco Digital Instruments, Santa Barbara, CA) was used for tapping mode AFM imaging using silicon probes with spring constant k = 305 kHz (OTESPA, Veeco). Topographic height and phase images were recorded simultaneously at 5126512 pixels at a scan rate of 0.4 Hz.

### Nanoparticle tracking analysis (NTA) for CT exosomes

Analysis of the absolute size distribution of the CT exosomes was performed using NanoSight NS300 (Malvern, UK). The light scattered by the exosomes with laser illumination was captured by a camera, and a video file of exosomes moving under Brownian motion was created. The NTA software tracked and analyzed particles individually from 10 to 1000 nm. Three recordings were performed for each sample.

### Isolation of total RNA from CT exosomes

Total RNA from CT-exos was extracted using a Total Exosome RNA and Protein Isolation Kit (cat. no. 4478545; Invitrogen). The freshly prepared CT-exos pellets were dissolved in 200 µL of exosome-resuspension buffer and loaded onto RNA columns according to the manufacturer's protocol. The RNA was finally eluted with 50 µL of RNase-free water. The quantity and quality of the RNA were determined using an Agilent 2200 TapeStation System High Sensitivity R6K Reagent (Agilent Technologies).

### Small RNA sequencing

Small RNAs were sequenced by the RiboBio Company (Guangzhou, China). Total RNA (10 µg) from the CT-exos was used to construct a library with the TruSeq® Small RNA Sample Prep Kit (Illumina, USA). Briefly, small RNAs (sRNAs) were recovered from a portion of the denaturing polyacrylamide gel corresponding to a size of approximately 18-30 nucleotides. The collected sRNAs were ligated with adapters at both ends. Subsequently, the adapter-ligated sRNAs were amplified by RT-PCR using low-cycle amplification. The PCR products were purified on a 12% polyacrylamide gel and the collected products were then sequenced using a HiSeq^TM^ 2500 instrument (Illumina, San Diego, CA, USA).

### Identification and quantification of miRNAs included in CT exosomes

The fifty-one-nucleotide raw reads obtained from sequencing were processed by removing poor quality reads, reads without a 3' adapter, 5' adapter- contaminated reads, reads without an insert fragment, reads containing poly (A) stretches, and reads shorter than 18 nt to acquire clean reads and generate the miRNA expression profile. The 18- to 30-nt modified sequences were mapped onto rat genome 5 using the Burrows-Wheeler Alignment Tool (BWA) and the default parameters. The clean reads were compared with small RNA databases (miRNA from miRBase20.0; rRNAs, tRNAs, snRNAs and snoRNA from Rfam11.0; piRNA from piRNAbank) to annotate the sRNA sequences. The clean reads with fewer than 10 copies were removed from the list. Only sRNAs whose precursor and mature sequences perfectly matched known rat miRNAs in the miRBase were considered conserved miRNAs. The expression level of miRNAs in the CT-exos was normalized using the following formula: transcripts per million (normalized expression (NE) = actual miRNA count/total clean read count ×1,000,000).

### Analysis of CT exosome uptake in CMECs

The conditioned medium from CTs (including secreted CT-exos; 10 mL) was collected as described above and then mixed with DiI (1,1'-dioctadecyl-3,3,3'3'-tetramethylindocarbocyanine perchlorate; 10 µM final concentration; cat. no. 60010; Biotium) for 15 min at room temperature, followed by dialysis for 24 h with 3 changes of 500 ml of PBS (pH 7.4) using dialysis tubing with a 2 kDa cutoff. After dialysis, the DiI-stained CT-exos were extracted with an ExoQuick^TM^ precipitation solution (cat. no. EXOTC50A-1; SBI). The collected exosomes were resuspended in 100 µL of PBS (pH 7.4). The isolated rat CMECs were prepared as described above, incubated with DiI-stained CT-exos (5 µL) for 6 h, and then incubated with LysoTracker (cat. no. L7526; Invitrogen) for 2 h in CMEC culture medium at 37 °C in a 5% CO_2_ and 95% air incubator. After staining with Hoechst 33342, the CT-exos-treated CMECs were imaged with a confocal microscope (FV3000, Olympus) using a 60 × oil-immersion objective.

### Transwell coculture assay for exosomes secreted by CTs

For the CT exosome transfer assay, transwell cocultures of CTs (upper chamber) and CMECs (lower chamber) were conducted using transwells with 0.4-μm-pore polyethylene terephthalate (PET) membranes (cat. no. PIHT30R48; Millipore), which allow exosomes to pass through but do not allow direct cell-to-cell contact between the upper and lower wells. CMECs (2×10^5^) were seeded in the lower chamber and cultured in CMEC culture medium at 37 °C in a 5% CO_2_ and 95% air incubator overnight. Then, the medium was replaced with medium 199 basic supplemented with 10% exosome-free fetal bovine serum. CTs (2×10^5^ cells) transfected with 100 nM miR-21-5p-FAM agomir for 6 h or stained with calcein-AM dye (cat. no. 40719ES50; Yeasen) for 30 min with extensive washing or staining with DiI dye (cat. no. 60010; Biotium) for 30 min with extensive washing were added to the upper chamber and cocultured for 48 h. The treated CMECs were imaged under microscopy (FV3000, Olympus) or washed with PBS (pH = 7.4) and then collected with 0.25% trypsin for fluorescence assessment using a flow cytometer (Cytoflex; Beckman Coulter). The CT-free cells in the upper chamber were used as a blank control.

For the CT endogenous exosomal miRNA-21-5p knockdown assay, similarly, transwell cocultures of CTs and CMECs were used. CMECs (2×10^5^) were seeded in the lower chamber and cultured in CMEC culture medium at 37 °C in a 5% CO_2_ and 95% air incubator overnight. Then, the medium was replaced with medium 199 basic supplemented with 10% exosome-free fetal bovine serum. CTs or CTs transfected with miR-21-5p antagomir (CTs-miR21KO) were seeded in the upper chamber for 72 h of coculture. The treated CMECs were collected to extract total RNA. The expression of miR-21-5p in cocultured CMECs was detected using real-time quantitative-PCR (qPCR). The CTs-free cells in the upper chamber were used as a blank control.

### CT exosome treatment of CMECs

For CT-exos treatment analysis, CMECs were cultured at a density of 5×10^4^ cells/mL and allowed to attach and grow overnight. The next day, they were treated with 300 μg/mL CT-exos or CT-exos-miR21KO for 24 h. A group treated with PBS in parallel was used as a control. The treated CMECs were used for q-PCR, cell viability assays, migration assays, tube formation assays, apoptosis assays and western blot assays.

### Cell viability assay

Cell viability was measured using the CCK-8 assay kit (cat. no. CK04; Dojindo) according to the manufacturer's instructions. Briefly, 10 µL of CCK-8 solution was added to each well (100 µL of medium) and incubated for 1 h at 37 °C in a 5% CO_2_ and 95% air incubator, and the absorbance was then measured at 450 nm in a microplate reader (Biotek). CMECs were cultured in CMEC culture medium at 37 °C in a 5% CO_2_ and 95% air incubator set as normoxia, while CMECs were cultured in serum-free medium 199 basic at 37 °C in a 5% CO_2_, 94% N_2_ and 1% O_2_ incubator set as ischemia-hypoxia. For analysis of the CT-exos effect, the CT-exos-, CT-exos-miR21KO- and PBS-treated groups were observed, while for analysis of the miR-21-5p effect, the miR-21-5p, scramble, CT-exos and blank groups were investigated. In addition, for analysis of the siCdip1 effect, the siCdip1, scramble and blank groups were observed.

### Migration assay

For the wound healing migration assay, CMECs were cultured on 24-well plates at a density of 1×10^5^ cells per well. The monolayer CMECs (approximately 80% confluence) were scratched with 1 mL pipette tips and washed with PBS to remove the cell debris. M199 basic supplemented with different types of exosomes (1 mL) or PBS was added to each well. Images were captured using an inverted phase microscope (Olympus IX71, Japan) at 0 h, 12 h and 24 h after the cell layer was wounded. The migration rate (%) was calculated as follows: migration rate (%) = (A0 - An)/A0 × 100%, where A0 represents the initial wound area (t = 0 h) and An represents the residual area at the measuring point (t = n h).

### Tube formation assay

The *in vitro* angiogenesis assay was conducted using Matrigel Basement Membrane Matrix (cat. no. 354234; Corning) according to the manufacturer's instructions. Briefly, the Matrigel was thawed overnight at 4 °C. Then, the Matrigel was added to 48-well plates at 150 µL per well using cold tips on ice and incubated at 37 °C to solidify. Next, CMECs (5×10^4^ cells per well) were resuspended in serum-free medium supplemented with different types of treatments and cultured on solidified Matrigel under normoxic and ischemia-hypoxic conditions. After 8 h of incubation, the cells were photographed using an inverted optical microscope (Olympus IX71, Japan), and the length of the formed tube was calculated using ImageJ 1.22 software (National Institutes of Health, USA). CMECs were cultured in CMEC culture medium at 37 °C in a 5% CO_2_ and 95% air incubator as the normoxic condition or in serum-free medium 199 basic at 37 °C in a 5% CO_2_, 94% N_2_ and 1% O_2_ incubator as the ischemic-hypoxic condition. For determination of the effect of CT-exos, the CT-exos-, CT-exos-miR21KO- and PBS-treated groups were observed, while for analysis of the miR-21-5p effect, the miR-21-5p, scramble, CT-exos and blank groups were investigated.

### Apoptosis assay using flow cytometry

CMECs were cultured in CMEC culture medium at 37 °C in a 5% CO_2_ and 95% air incubator as the normoxic condition or in serum-free medium 199 basic at 37 °C in a 5% CO_2_, 94% N_2_ and 1% O_2_ incubator as the ischemic-hypoxic condition. For analysis of the CT-exos effect, the CT-exos-, CT-exos-miR21KO- and PBS-treated groups were observed, while for analysis of the miR-21-5p effect, the miR-21-5p, scramble, CT-exos and blank groups were investigated. In addition, for analysis of the siCdip1 effect, the siCdip1, scramble and blank groups were observed. An Annexin V/PI kit (FXP023-100, 4A Biotech) and subsequent flow cytometry analysis were applied to investigate the apoptosis of treated CMECs according to the manufacturer's instructions. Briefly, the treated CMECs (5×10^5^) were washed with cold PBS, resuspended in 100 µL of binding buffer and stained with 5 µL of Alexa Fluor 647-conjugated Annexin V. Following a 5-min incubation in a dark room, 10 µL of PI and 400 µL of binding buffer were added. Finally, the cells were analyzed using a flow cytometer (Cytoflex; Beckman Coulter). Early and late apoptotic cells were examined using plots of fluorescence 2 (FL2 for PI) versus fluorescence 1 (FL1 for Annexin V). A total of 10,000 events were collected and analyzed for each sample.

### Transfection of rno-miR-21-5p mimics in CMECs

The isolated CMECs were cultured to 80% confluence with complete culture medium as described above at 37 °C in a 5% CO_2_ and 95% air incubator and then transfected with rno-miR-21-5p mimics (5'-UAGCUUAUCAGACUGAUGUUGA-3'; 100 nM; GenePharma) or the scrambled control (5'-UUCUCCGAACGUGUCACG-3'; 100 nM; GenePharma) using Lipofectamine RNAiMAX (cat. no. 13778150; Invitrogen). The transfected cells were used for qPCR, cell viability assays, migration assays, tube formation assays, apoptosis assays or western blot assays. In the above assays, 3 identical wells were observed in each analysis, and three repeat experiments were conducted.

### Prediction of microRNA-targeted genes

TargetScan (http://www.targetscan.org/) and miRWalk (http://www.umm.uni-heidelberg.de/apps/zmf/mirwalk/index.html) were used to predict the potential target genes of rno-miR-21-5p. Only genes predicted by both databases were used for the subsequent functional analysis.

### Dual luciferase reporter assay

Luciferase reporters were constructed by cloning sequences from the 3' untranslated regions (3'UTRs) of the *Cdip1* mRNAs into the psiCHECK-2 vector (cat. no. C8021, Promega). Briefly, the wild-type 3' UTR of the *Cdip1* gene, which contains the predicted miR-21 response element, as well as mutant 3' UTRs were synthesized and then inserted in the multiple cloning region behind the synthetic *Renilla* luciferase gene. HEK293 cells were cotransfected with the constructed reporter plasmid (0.05 μg) and rno-miR-21-5p mimics (50 nM) or the scrambled control using Lipofectamine 3000 transfection reagent (cat. no. L3000008, Invitrogen). A reporter assay was performed 48 h after transfection using the Dual Luciferase Reporter Assay System (cat. no. E1910, Promega) according to the manufacturer's protocol. The luciferase signal was normalized to the signal arising from an intra plasmid Renilla/firefly luciferase transfection reporter cassette in HEK293 cells.

### Isolation of total RNA and real-time quantitative PCR

Total RNA was extracted using TRIzol reagent (cat. no. 15596018; Invitrogen) according to the manufacturer's protocol. The quantitative analysis of rno-miR-21-5p expression was performed with a Hairpin-it miRNA q-PCR Quantitation Kit (cat. no. E01006-E01015; GenePharma) according to the manufacturer's instructions. First, the extracted RNA (1 μg) was reverse-transcribed using specific stem-loop primers as described in the manual, and then, the expression levels of rno-miR-21-5p were analyzed using SYBR Green-based real-time PCR. The reaction mixture was composed of 10 µL of 2× Real-time PCR buffer, 0.32 µL of miR-21-5p specific primers, 0.2 µL of Taq DNA polymerase, 7.48 µL of PCR-grade water and 2 µL of the cDNA template. Amplifications were performed on a Mini-Opticon System (Bio-Rad) using the following PCR conditions: initial denaturation at 95 °C for 3 min followed by 40 cycles of amplification at 95 °C for 12 s and 62 °C for 40 s. Ct values were averaged and normalized to U6. Relative expression was determined using the 2^-ΔΔCt^ comparative threshold method.

For analysis of the expression of the *phosphatase and tensin homolog* (*Pten*),* programmed cell death programmed cell death 4* (*Pdcd4*), and* Cdip1* genes, the extracted RNA (1 μg) was reverse-transcribed into first-strand cDNAs using ReverTra Ace q-PCR RT Master Mix with gDNA Remover (cat. no. FSQ-301, Toyobo) according to the manufacturer's instructions, and then, the gene expression levels were analyzed using SYBR Green-based real-time PCR. The reaction mixture was composed of 10 µL of SYBR Green PCR Master Mix (cat. no. B21202; Biotool), 1 µL of each primer, 8 µL of PCR-grade water and 1 µL of the cDNA template. The following primers were used (5'-3'): *Pten* forward: GGGAAAGGACGGACTGGTGT, reverse: ATAGCGCCTCTGACTGGGAAT; *Pdcd4* forward: GAGCACGGAGATACGAACGAA, reverse: GTCCCGCAAAGGTCAGAAAG; and *Cdip1* forward: GACTTCAGCCTTTTGTTCATGG, reverse: TCTTTGCTGTTGATACTCCTGG. Amplifications were performed with a Mini-Opticon System using the following conditions: 95 °C for 5 min, followed by 40 cycles of denaturation at 95 °C for 15 s and primer annealing and extension at 62 °C for 30 s. All cDNA samples were amplified in triplicate and normalized to β-actin expression on the same plate. Three replicates of each specimen were examined. The results of this study were generated from three rats.

### RNA interference

The synthesized si-*Cdip1* (si*Cdip1*: 5'-CCUACAUGCCUGCAGGUUUdTdT-3') and scrambled control (as described above) (GenePharma, China) were transfected into CMECs with Lipofectamine RNAiMAX (cat. no. 13778150; Invitrogen), according to the manufacturer's instructions. Briefly, CMECs were cultured for 12 h with CMEC culture medium as described above at 37 °C in a 5% CO_2_ and 95% air incubator. Lipofectamine RNAiMAX was mixed with siRNA (100 nM) or the scrambled control, added to the cells and incubated at 37 °C for 24 h to silence the RNA. CMECs were incubated with the transfection complex for 48 h, incubated in the ischemia-hypoxia environment for 24 h and then harvested for flow cytometry analysis using the Annexin V/PI assay or western blot analysis.

### Western blot analysis

The CT-exos or CMEC lysates were prepared in radioimmunoprecipitation assay (RIPA) buffer (cat. no. P0013C; Beyotime) containing a protease inhibitor cocktail (cat. no. W2200s; Cwbio). The total protein concentration was analyzed using a BCA Protein Assay Kit (cat. no. P0010; Beyotime). The extracted CT-exos or CMEC proteins were denatured in loading buffer (5× SDS-PAGE loading buffer: 0.5 M Tris-HCl, pH 6.8, 0.05% beta-mercaptoethanol, 1% SDS, 0.005% bromophenol blue, 50% glycerol) for 10 min at 95 °C, electrophoresed on a 12% SDS-PAGE gel, and transferred to a PVDF membrane (cat. no. 162-0177; Bio-Rad). After being blocked with 5% nonfat milk in TBST buffer, the PVDF membranes were incubated with the appropriate primary antibodies, including anti-CD63 (1:1000; cat. no. EXOAB-CD63A-1; SBI), anti-Cdip1 (1:1000; cat. no. NBP1-76996; Novus), anti-cleaved Caspase-3 (1:1000; cat. no. 9662S; CST), anti-β-actin (1:20000; cat. no. 66009-1-Ig; Proteintech), and anti-GAPDH (1:20000; cat. no. 60004-1-Ig; Proteintech) overnight, washed with TBST, and incubated with horseradish peroxidase (HRP)-conjugated secondary antibodies. The protein bands were visualized and imaged using an enhanced chemiluminescence system on a chemiluminescence reader (GeneGnome HR; Synoptics).

### Induction of myocardial infarction and intramyocardial injection

MIs were generated in three-month-old female SD rats as our and others previously described 19, 20, 28, 30-32. Briefly, the rats were anesthetized with ketamine (100 mg/kg, i.p.) and underwent a left intercostal thoracotomy. The left anterior descending coronary artery (LAD) was identified and then ligated directly below the left atrial appendage with 8-0-gauge nylon sutures. The presence of pallor and abnormal movement of the left ventricle confirmed LAD occlusion. Intramyocardial injections were performed within 30 min after LAD ligation. CTs, CT-exos (300 µg of total protein in 50 µL of PBS [pH 7.4]) and the rno-miR-21-5p agomir (50 µg in 50 µL of RNase-free water; chemically modified and designed for *in vivo* study) were injected using a 30-gauge needle and a 10-µL Hamilton syringe. Each heart received 5 injections (10 µL/injection), with 3 injections in border areas of the ischemic zone and 2 injections in the center of the ischemic area. Rat models of MI were injected with PBS (pH 7.4; 50 µL) and served as a control for CT or CT-exos treatment. One group injected with scrambled mimics (50 µg in 50 µL of RNase-free water) served as a microRNA control. The chest wall was then closed, the lungs were inflated, the rat was extubated, and the thoracotomy was closed. After recovery, the rats were returned to the animal facility. The groups receiving CTs, CT-exos, PBS and sham were evaluated for cardiac function and anesthetized on day 28, and the groups receiving the miR-21-5p agomir and scrambled control were evaluated for cardiac function and anesthetized on day 7 to collect the treated hearts. The tissues were fixed with 4% paraformaldehyde, embedded in paraffin wax and sectioned. In the present study, for the studies of CTs and CT-exos treatment, the animal numbers used for surgery in the CTs, CT-exos, PBS-1 (control for CTs), PBS-2 (control for CT-exos) and sham groups were 14, 9, 14, 5 and 5, respectively. The survival rates of the CTs, CT-exos, PBS-1, PBS-2 and sham groups were 35.7% (5/14), 11.1% (1/9), 35.7% (5/14), 0% and 0%, respectively. As one animal in the CTs group, two animals in the CT-exos group and one animal in PBS-2 failed to undergo LAD ligation, the numbers in the CTs, CT-exos, PBS-1, PBS-2 and sham groups for final analysis were 8, 6, 9, 4 and 5, respectively. For the study of miR-21-5p treatment, the number of animals used for surgery in the miR-21-5p-, scramble and sham groups was 7, 7 and 5, respectively. The survival rates of the miR-21-5p-, scramble- and sham-treated groups were 14.3% (1/7), 14.3% (1/7) and 0%, respectively.

### Echocardiography

Transthoracic echocardiograms were performed on experimental rats. The experimental rats were anesthetized with ketamine (100 mg/kg, i.p.). The echocardiographic measurements were then collected using an Acuson Sequoia 256c ultrasound system equipped with a 13-MHz linear transducer from a Vevo 2100 echocardiogram (VisualSonics, Canada). Briefly, the anterior chest wall was shaved, and the rat was placed in a left lateral decubitus position. A rectal temperature probe was inserted, and the body temperature was carefully maintained between 37 °C and 37.5 °C on a heating pad throughout the study. Parasternal long-axis, parasternal short-axis and 2 apical four-chamber views were collected in 2D-M-mode. The systolic and diastolic anatomic parameters were obtained from M-mode tracings at the mid-papillary level. The ejection fraction (EF) was calculated using the area-length method 19, 20.

### Histological analysis

The extent of MI was measured at the level of the mid-papillary heart muscles and scored following Masson's trichrome staining. Paraffin sections were dewaxed and rinsed with water using routine protocols. After iron hematoxylin staining (7 min), the sections were stained with Ponceau acid fuchsin (5 min) and rinsed with distilled water. After differentiation using a phosphomolybdic acid solution (5 min), the sections were sequentially stained with aniline blue (5 min) and 1% acetic acid (1 min). After staining, the sections were dehydrated, cleared in xylene, and then mounted with resinene.

The infarct size, with linear approximations to account for area gaps in histology, is expressed as a percentage of the total LV myocardial area as our and others previously described 19, 20, 28, 30-32. The collagen area of the infarct zone (CAIZ) is expressed as the ratio of the area of blue staining in the infarct zone to staining in the noninfarct zone using Image-Pro Analyzer 6.0 software. The perivascular collagen volume area (PVCA) is expressed as the ratio of the peripheral collagen area surrounding the small artery to the artery lumen area. The small arteries were selected for a detailed analysis at 40 × magnification, and the average was used as the definitive PVCA.

### Immunohistochemistry and TUNEL staining

Blood vessel density in the infarct zone and border zone was measured using immunostaining for vWF. Immunohistochemical staining was performed on heart tissue samples using a rabbit vWF antibody (1:300; cat. no. F3520; Sigma), peroxidase-conjugated goat anti-rabbit IgG (cat. no. SA00001-2; Proteintech) and a DAB-Plus Staining Kit (cat. no. 00-2020; Life Technologies). The vWF^+^ blood vessels present throughout the infarct zone and border zone were photographed and counted under a microscope (20× magnification). The number of vessels per mm^2^ was compared among the different groups. The measurements were conducted in a double-blinded manner by two independent investigators.

The apoptotic endothelial cells were evaluated by TUNEL double staining (cat. no. KGA7063; KeyGene Biotech) and immunostaining for vWF (cat. no. F3520; Sigma) in the infarct zone 1 week after MI according to the manufacturer's protocols. Briefly, the paraffin sections at the mid-papillary level of the hearts were pretreated with 15 μg/ml proteinase K diluted with 10 mM Tris/HCl buffer (pH 7.4) for 15 min. After being rinsed with PBS, the sections were incubated with 50 μL of TUNEL reaction mix at 37 °C for 60 min. Next, the sections were incubated with 50 μL of streptavidin-TRITC labeling solution for 30 min at 37 °C. Then, the sections were blocked with 1% BSA for 1 h and incubated with anti-vWF (1:300) overnight at 4 °C. After the sections were washed, they were incubated with Alexa Fluor 488-conjugated secondary antibodies (1:1000) for 1 h and then incubated with Hoechst nuclear counterstain. The vWF^+^ and TUNEL^+^ cells were photographed and counted using confocal microscopy (FV3000, Olympus). The number of apoptotic endothelial cells per mm^2^ was compared among the different groups.

### Statistical analysis

An independent samples t-test and one-way ANOVA with least significant difference (LSD) test were performed using SPSS version 17 software to determine the *P*-values in repeated experiments. All values are expressed as the mean± standard deviation (S.Dev). *P* < 0.05 was considered to indicate statistically significant differences.

## Results

### Transplantation of CTs improved cardiac function, decreased infarct size and fibrosis, and enhanced angiogenesis

We isolated CTs according to the protocol described previously 19, 20. The CTs (Figure S1) in this study showed the commonly accepted morphology and molecular marker features of telocytes 21-26. These cells were more similar to interstitial cells than endothelial cells and progenitor stem cells. This deduction is supported by the following findings included in Figure S1: 1) the cells were positive for c-Kit, CD34 and Vimentin but negative for the endothelial cell markers CD31 and vWF (Figure S1B). 2) Cultured CTs did not show colony formation (Figure S1C) and entered growth arrest at approximately 10-15 passages when they were serially subcultured (data not shown). In addition, the collected CTs (passages 1~2) failed to be induced to differentiate into mesoderm-derived cells, such as bone and fat cells (Figure S1D). 3) The preliminary results of single-cell RNA sequencing revealed that the collected CTs were negative for the common stem cell markers Oct-4, SSEA-1 and Sca-1. In addition, they were positive for CD105, CD73 and Col1a1 (Figure S1E), which are commonly expressed by mesenchymal linage-derived interstitial cells.

**Figure 1:**
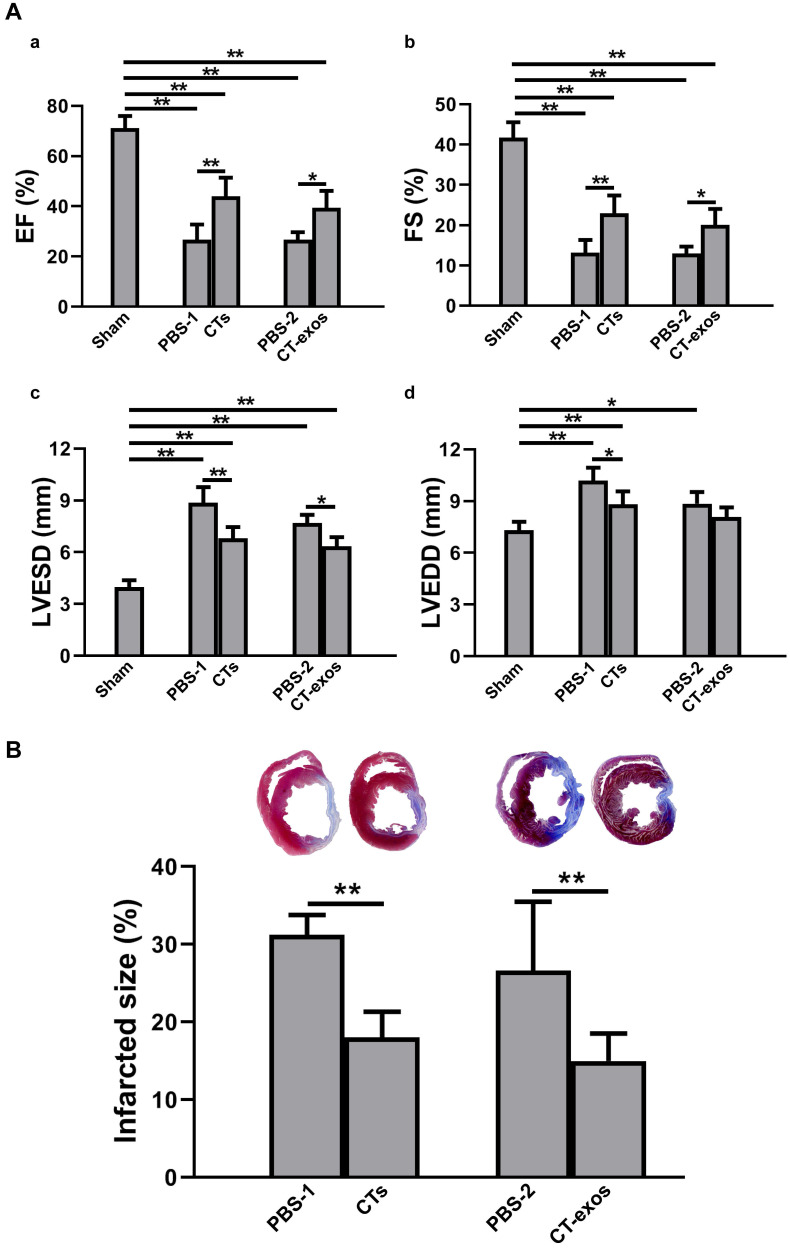
**Transplantation of CTs and CT-exos improved cardiac function and decreased infarct size. A:** Echocardiography revealed that transplantation of CTs significantly improved the (a) ejection fraction and (b) fractional shortening and significantly reduced the final (c) systolic as well as (d) diastolic diameter compared with those of the PBS-1 group. n = 5, 9, and 8 for the sham, PBS-1, and CT groups. In addition, echocardiography revealed that cardiac function measurements, including (a) ejection fraction, (b) fractional shortening, and (c) final systolic diameter but not (d) final diastolic diameter, of the CT-exos groups were significantly superior to those of the PBS-2 group. n = 5, 4, and 6 for the sham, PBS-2, and CT-exos groups. **B:** The infarct size of the CTs group was significantly smaller than that of the PBS group. n = 8, and 8 for the PBS-1, and CT groups. In addition, the infarct size of the CT-exos group was significantly smaller than that of the PBS-2 group. n = 4, and 5 for the, PBS-2 and CT-exos groups. *: *p* < 0.05, **:* p* < 0.01.

**Figure 2:**
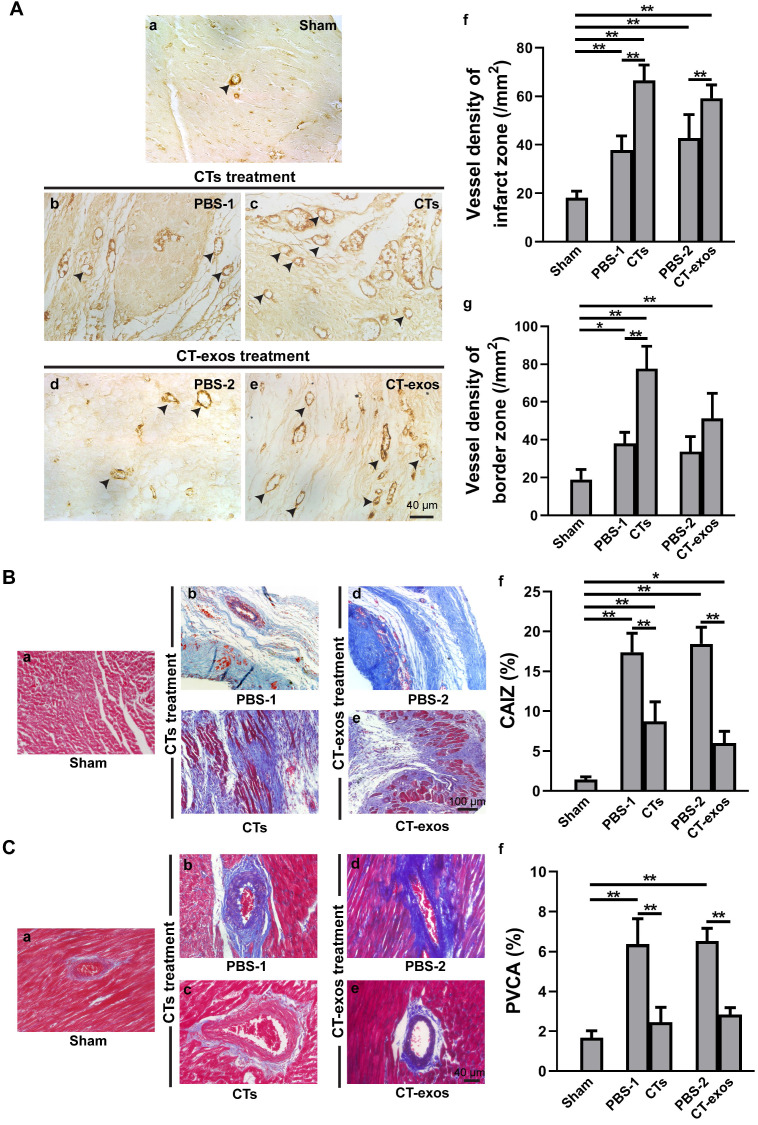
** Transplantation of CTs and CT-exos increased angiogenesis and decreased fibrosis. A:** The vessel density (vWF^+^) in the infarct and border zones of the CT group was significantly increased compared with that in the PBS group. n = 5, 6, and 6, for the sham, PBS-1, and CT groups. In addition, the vessel density (vWF^+^) in the infarct zone, but not the border zone, was significantly increased in the CT-exos group compared with the PBS group. n = 5, 4, and 5 for the sham, PBS-2, and CT-exos groups. Sham group (a). PBS-1-treated infarct zone (control for CT treatment) (b). CT-treated infarct zone (c). PBS-2-treated infarct zone (control for CT-exos treatment) (d). CT-exos-treated infarct zone (e). Semiquantitative analysis of the infarct zone (f). Semiquantitative analysis of the border zone (g). **B:** The collagen area of the infarct zone (CAIZ) of the CT group (a-c, f) and CT-exos group (a, d, e, f) was significantly smaller than those of the PBS-1 group and the PBS-2 group respectively. n = 5, 7, and 7 for the sham, PBS-1, and CTs groups. n = 5, 4, and 5 for sham, PBS-2, and CT-exos groups. Sham group (a). PBS-1-treated infarct zone (b). CT-treated infarct zone (c). PBS-2-treated infarct zone (d). CT-exos treated infarct zone (e). Semiquantitative analysis for a-e (f). **C:** The the perivascular collagen volume area of the noninfarct zone (PVCA) of the CT group (a-c, f) and CT-exos group (a, d, e, f) was significantly smaller than those of the PBS-1 group and the PBS-2 group respectively. n = 5, 7, and 7 for the sham, PBS-1, and CTs groups. n = 5, 4, and 5 for sham, PBS-2, and CT-exos groups. Sham group (a). PBS-1-treated noninfarct zone (b). CT-treated noninfarct zone (c). PBS-2-treated noninfarct zone (d). CT-exos treated noninfarct zone (e). Semiquantitative analysis for a-e (f). *: *p* < 0.05, **:* p*< 0.01.

To test the therapeutic effects of these cells on MI, we injected the isolated CTs (5 × 10^5^ cells) into the infarcted site and the border zone after MI caused by LAD ligation. Four weeks after the operation and CT transplantation, echocardiography showed that both critical indicators reflecting cardiac function, ejection fraction (EF) and fractional shortening (FS), were significantly improved in the CTs-treated group (Figure 1A a&b). Consistently, two geometrical parameters, left ventricular end-systolic diameter (LVESD) and left ventricular end-diastolic diameter (LVEDD), were significantly decreased in the CTs-treated group, indicating a recovery in ventricular filling and pumping function (Figure 1A c&d). Consistent with our previous reports 19, 20, injection of CTs significantly decreased infarct size (Figure 1B) and increased the density of the vWF^+^ vessels in the infarct and border zones (Figure 2A a-c, f, g). The collagen area of the infarct zone (CAIZ) (Figure 2B a-c, f) and the perivascular collagen volume area of the noninfarct zone (PVCA) (Figure 2C a-c, f) were likewise reduced. These observations demonstrated that transplantation of CTs can improve cardiac function and angiogenesis as well as reduce fibrosis and infarct size after MI.

### The therapeutic effects of CT therapy following MI were mediated by CT-derived exosomes that facilitate cardiac angiogenesis

**Figure 3:**
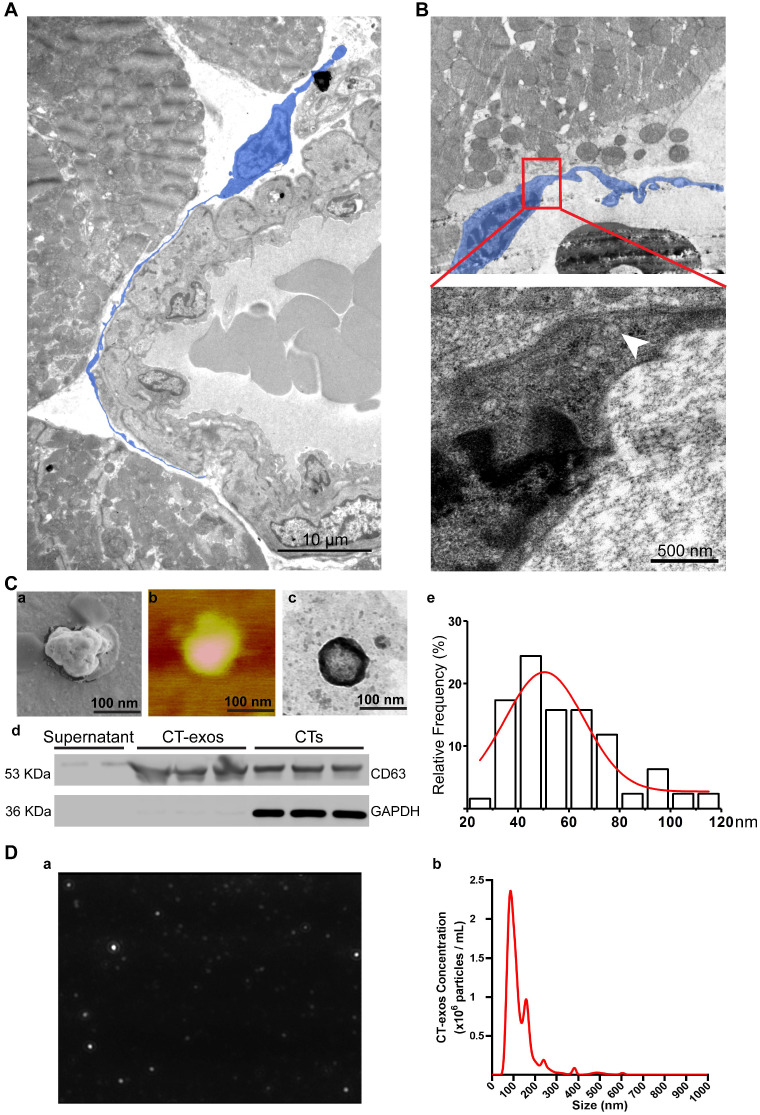
** Cardiac telocytes present a unique morphology and secreted exosomes. A:** Transmission electron microscopy images of CT (blue) in the myocardium show a unique morphology: a piriform, spindle or triangular shape with a nucleus that occupies approximately 25-30% of the cell volume and very long, thin, dichotomously branched protrusions with a moniliform aspect called telopods. **B:** CTs secrete exosomes (white arrow). **C:** (a-c) Scanning electron microscopy, atomic force microscopy and transmission electron microscopy images of the collected CT-exos confirm that the morphology and diameter are consistent with exosomes. (d) Western blots confirmed that the collected CT-exos are positive for CD63. (e) Relative frequency analysis of the diameter of the CT-exos. **D:** Nanoparticle tracking analysis (NTA) of the CT-exos. (a) Representative NTA image of the CT-exos. (b) Relative frequency analysis of the diameter of the CT-exos by NTA. The collected vesicles were confirmed to be exosomes by the above evidence.

As promoting angiogenesis and reconstructing the vessel network in ischemic myocardium is still a major challenge for the regeneration of MI, our present study aimed to identify possible mechanisms underlying the increase in angiogenesis. Consistent with the observation made by Popescu's group 33, our TEM studies also showed that CTs do exist in rat myocardium and showed unique morphology: a piriform, spindle or triangular shape with a nucleus that occupies approximately 25-30% or even more of the cell volume and very long, thin, dichotomously branched protrusions with a moniliform aspect called telopods, which are considered the 'ultrastructural hallmark' that distinguishes CTs from other interstitial cells (such as cardiac fibroblasts) 21-26 **(**Figure 3A). Moreover, we observed that CTs in rat myocardium shed exosomes (Figure 3B). These findings indicate that CT communication with CMECs to enhance the angiogenic potential of CMECs *via* CT-exos could be an endogenous function in the myocardium as well as one of the reasons for the increase in cardiac angiogenesis and the regenerative effects of CT therapy. Accordingly, CT-exos were collected to examine this hypothesis. The collected vesicles were confirmed to be exosomes by SEM (Figure 3C a), AFM (Figure 3C b), TEM (Figure 3C c), western blot analysis (Figure 3C d), dimension analysis (Figure 3C e), and NTA (Figure 3D and Supplementary video).

To determine the biological activity of the CT-exos, we used the same injection and analysis protocol described for the CTs to determine the therapeutic activity of the CT-exos in MI. The ability of the CT-exos to promote angiogenesis in ischemic myocardium was determined based on a significant increase in the density of vWF^+^ vessels in the infarct zone, but not in the border zone, compared with that in the PBS control group (Figure 2A a, d, e, f, g). In addition, the infarct size and related fibrosis (CAIZ and PVCA) were significantly reduced in the CT-exos-treated group compared with the control group (Figure 1B; Figure 2B a, d, e, f and Figure 2C a, d, e, f). Correspondingly, the cardiac function revealed by the echocardiography and geometrical parameters was significantly improved, which was supported by the cardiac function measurements, including (a) ejection fraction, (b) fractional shortening, and (c) final systolic diameter but not (d) final diastolic diameter, of the CT-exos groups; these parameters were significantly superior to those of the PBS group (Figure 1A). In summary, CT-exos possess the same therapeutic effects as CTs to promote regeneration following MI by increasing angiogenesis, decreasing the infarct as well as fibrosis size, and improving cardiac function when they were transplanted into the ischemic myocardium.

### CT exosomes internalized by CMECs promoted the survival and inhibited the apoptosis of CMECs grown under ischemic-hypoxic conditions *in vitro*

As CTs improved angiogenesis and reduced infarct size, the potential of CMECs to serve as recipient cells of CT-exos was investigated *in vitro*. CT-exos were first labeled with DiI and then cocultured with CMECs for 6 h. Thereafter, the CMECs were labeled with LysoTracker (a probe for lysosomes) for 2 h. After this treatment, many DiI-labeled exosomes were detected in the cytoplasm (red fluorescence; Figure S2A a-c) and had not been transported to the lysosomes (green fluorescence; Figure S2A d). Thus, most of the CT-exos were internalized into CMECs but not targeted for early lysosomal degradation, at least within 8 h after engulfment. In addition, a transwell assay between CTs and CMECs demonstrated that the CT-derived exosomes can be secreted, targeted and engulfed by CMECs. For confirmation of this finding, CTs with extensive washing after they were stained by calcein (green) and Dil (red) were added in the upper chamber and cocultured in the lower chamber, which was seeded with CMCEs, for 48 h. It was found that approximately 95% and 51% of the CMCEs were green and red, respectively, in the lower chamber by flow cytometry (Figure S2B a-c; Figure S2C a-c). In parallel, scattered green and red fluorescent dots were found in the treated CMCEs of the lower chamber by microscopy (Figure S2B d; Figure S2C d).

**Figure 4:**
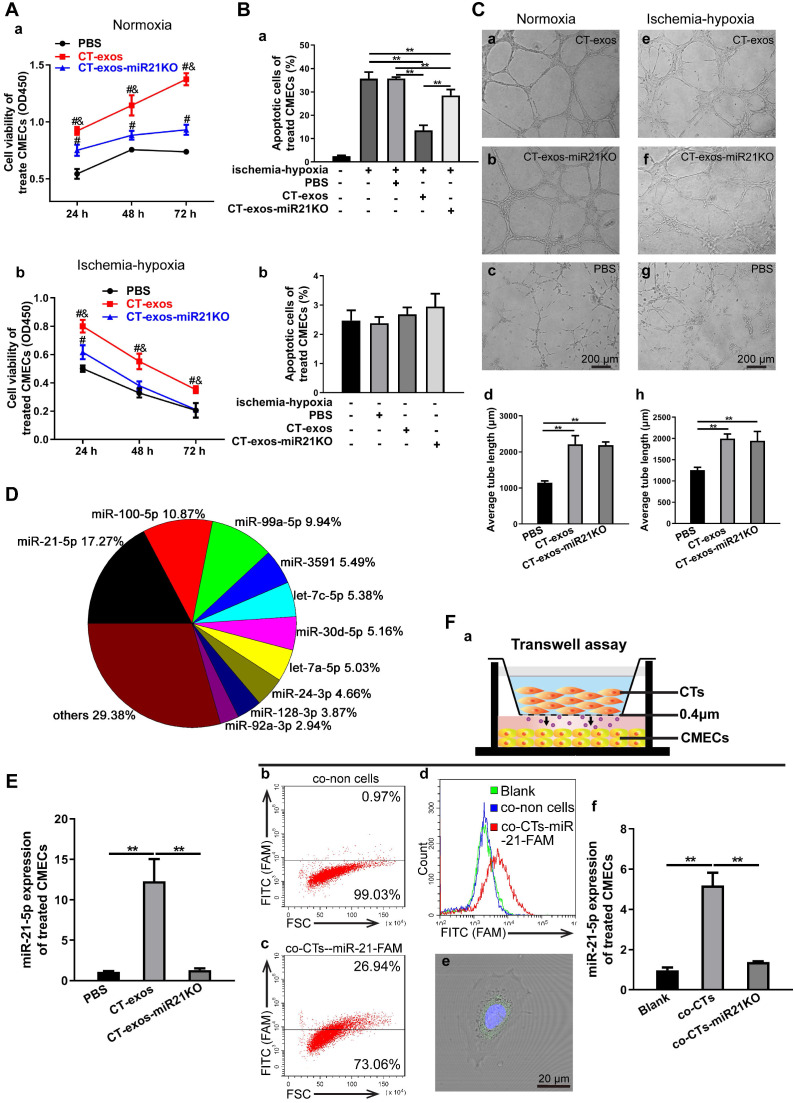
** CTs exosomes facilitated survival and inhibited apoptosis in CMECs and enhanced angiogenesis in ischemic-hypoxic conditions *in vitro*, and CT-miR-21-5p was identified as a key functional molecule. A:** The cell viability of CMECs incubated with CT-exos, CT-exos-miR21KO or PBS under normoxia and ischemia-hypoxia. **B:** Annexin V/PI staining and flow cytometry of CT-exos-, CT-exos-miR21KO-, PBS- or blank-treated CMECs cultured under normoxic and ischemic-hypoxic conditions for 24 h. **C:** Tube formation assay of CMECs incubated with CT-exos, CT-exos-miR21KO or PBS under normoxic and ischemic-hypoxic conditions for 8 h. The above studies (A-C) were conducted using two animals in at least three repeated experiments for each individual animal. **D:** The 10 most abundant miRNAs in CT-exos according to RNA sequencing, and miRNA-21-5p is the most prevalent miRNA. **E:** miR-21-5p expression levels in CMECs incubated with PBS, CT-exos or CT-exos-miR21KO for 24 h. **F:** (a) Schematics of the transwell assays of CT-exos for CMECs. (b) Flow cytometry analysis for CMECs which was cocultured with no CTs in the upper chamber for 48 h. (c) Flow cytometry analysis for CMECs (lower chamber) cocultured with CTs transfected by the synthesized miR-21-5p-FAM (green) in the upper chamber for 48 h. The green-stained CMCEs in the lower chamber comprised approximately 27%. (d) The green-stained CMCEs were only found in miR-21-5p-FAM (green)-CT treated group. (e) The scattered green dots were found in the miR-21-5p-FAM (green)-CT-treated CMCEs by microscopy. (f) The qPCR assay for transwell treatment confirmed that the miR-21-5p level of CT-treated CMCEs was significantly higher than that of the blank control, while the miR-21-5p level of the miR21KO-CT-treated CMCEs was similar to that of the blank control. *: *p* < 0.05, **: *p* < 0.01, #: *p* < 0.05 compared with the PBS group, &: *p* < 0.05 compared with the CT-exos-miR21KO group.

The protective effect of CT-exos on CMEC survival under normoxia and ischemia-hypoxia was further investigated by CCK-8 assays. The CCK-8 values of the CT-exos-treated CMECs under both normoxia and ischemia-hypoxia were significantly higher than those of the PBS-treated controls (Figure 4A). In addition, the apoptosis and cell death of the CT-exos-treated CMECs under ischemic-hypoxic conditions, revealed by Annexin V/PI flow cytometry assays, was significantly reduced compared with that of the PBS-treated controls, but these results were not found under normoxia (Figure 4B and Figure S3A). Furthermore, wound healing assays showed that the migration distance of CT-exos-treated CMECs was significantly longer than that of the PBS-treated CMECs 24 h after treatment (Figure S3B). In addition, the tube formation assay demonstrated that the tubular network of the tube formed for the CT-exos-treated CMECs in both normoxic and ischemic-hypoxic conditions was significantly better than those of the PBS-treated CMECs (Figure 4C). Taken together, the results indicate that the CMECs were recipient cells for CT-exos. After uptake into CMECs, CT-exos may remain in the cytoplasm to maintain the survival, migration, and tube formation of CMECs under normoxia and exert antiapoptotic and proangiogenic effects under ischemic-hypoxic conditions.

### miRNA-21-5p included in CT exosomes was identified as a key functional molecule that facilitated survival and inhibited apoptosis in CMECs and enhanced angiogenesis and regeneration following MI

**Figure 5:**
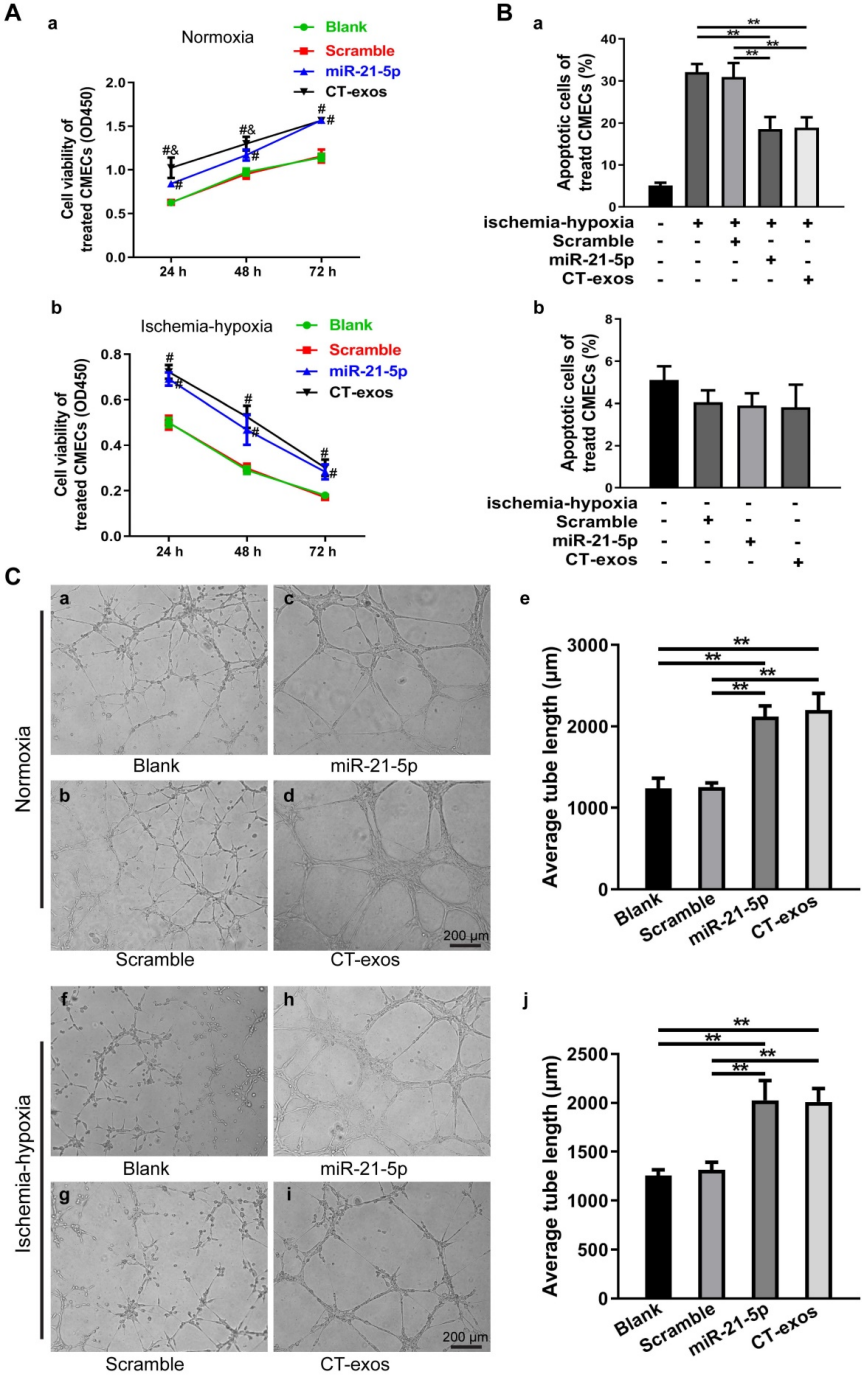
** miRNA-21-5p mimics facilitated survival and inhibited apoptosis in CMECs and enhanced angiogenesis *in vitro*. A:** The cell viability of CMECs transfected with miR-21-5p mimics, scramble, CT-exos or blank under normoxic and ischemic-hypoxic conditions. **B:** Annexin V/PI staining and flow cytometry assay of miR-21-5p mimic-, scramble-, CT-exos-, and blank-treated CMECs cultured under normoxic and ischemic-hypoxic conditions for 24 h. **C:** Tube formation assay of CMECs transfected with miR-21-5p mimics, scramble, CT-exos and blank. The above studies (A-C) were conducted using two animals in at least three repeat experiments for each individual animal. **:* p*< 0.01, #: *p* < 0.05 compared with the blank group and scramble group, &: *p* < 0.05 compared with the miR-21-5p group.

Exosomes have recently been shown to fuse with live cells upon their release into the extracellular environment in both a paracrine and endocrine manner, which promotes the transfer of their cargo of proteins, lipids or RNAs to the target cells and plays key roles in intercellular signaling and communication 34. Accordingly, the miRNA profile of CT-exos was investigated using small RNA sequencing. The most abundant miRNA, miR-21-5p, which accounted for 17.2% of CT exosomal microRNAs, and the other top 10 listed miRNAs are shown in Figure 4D. To determine which of the above-identified effects of CT-exos were attributable to miR-21-5p, we used qPCR to confirm the expression of miR-21-5p in the CMECs treated with CT-exos or CT-exos-miR21KO, in which CT-exos miR-21-5p was knocked down significantly by treatment with antagomir miR-21-5p inhibitor (Figure S5A). The miR-21-5p expression of the CT-exos-treated CMECs was increased significantly compared with that of the PBS control, while the miR-21-5p expression following CT-exos-miR21KO treatment was similar to that of the PBS control (Figure 4E). Importantly, the transwell assay with CTs transfected with synthesized miR-21-5p-FAM (green; top) and CMECs (bottom) demonstrated that after coculture for 48 h, approximately 27% green-stained CMCEs in the lower chamber were identified by flow cytometry (Figure 4F a-c). In addition, green-stained CMCEs were only found in the miR-21-5p-FAM (green)-CT-treated group (Figure 4F d). In parallel, scattered green dots were found in the miR-21-5p-FAM (green)-CT-treated CMCEs by microscopy (Figure 4F e). Moreover, the qPCR assay for transwell treatment confirmed that the miR-21-5p level of the CT-treated CMCEs was significantly higher than that of the PBS control, while the miR-21-5p level of the miR-21KO-CT-treated CMCEs was similar to that of the PBS control (Figure 4F f). The above findings clearly demonstrated that CT-exosomal-miR-21-5p can be transferred to CMECs *via* the uptake of the CT-exos. Then, CT-exos-miR21KO treatment was used assessed by CCK-8, Annexin V/PI flow cytometry, wound healing and tube formation assays to compare with the effects of the CT-exos under normoxic and ischemic-hypoxic conditions. The effects of CT-exos-miR21KO treatment of CMECs on survival in normoxia and ischemia-hypoxia (Figure 4A), apoptosis inhibition in ischemia-hypoxia (Figure 4B) and migration promotion in normoxia (Figure S3B) were significantly poorer than those of the CT-exos treatment of CMECs, although this was not observed with tube formation under normoxia and ischemia-hypoxia (Figure 4C). Thus, the effects identified in the above studies were attributed to miR-21-5p inclusion in CT-exos. Indeed, our *in vitro* studies using CCK-8, Annexin V/PI flow cytometry, and tube formation assays under normoxic and ischemic-hypoxic conditions, as well as wound healing under normoxic conditions, further demonstrated that miR-21-5p treatment could significantly improve CMEC survival (Figure 5A), repress apoptosis of CMECs (Figure 5B and Figure S4A), and promote migration (Figure S4B) and tube formation (Figure 5C) of CMECs compared to these effects in the scramble-treated CMECs. In addition, the miR-21-5p-treated effects for the above parameters were similar to those of CT-exos treatment (Figure 5A-C and Figure S4A, B). All these findings indicated that miRNA-21-5p is a key functional molecule that mimics the effect of CT exosomes on survival, apoptosis and angiogenesis in cardiac endothelial cells under normoxic and ischemic-hypoxic environments *in vitro*.

**Figure 6:**
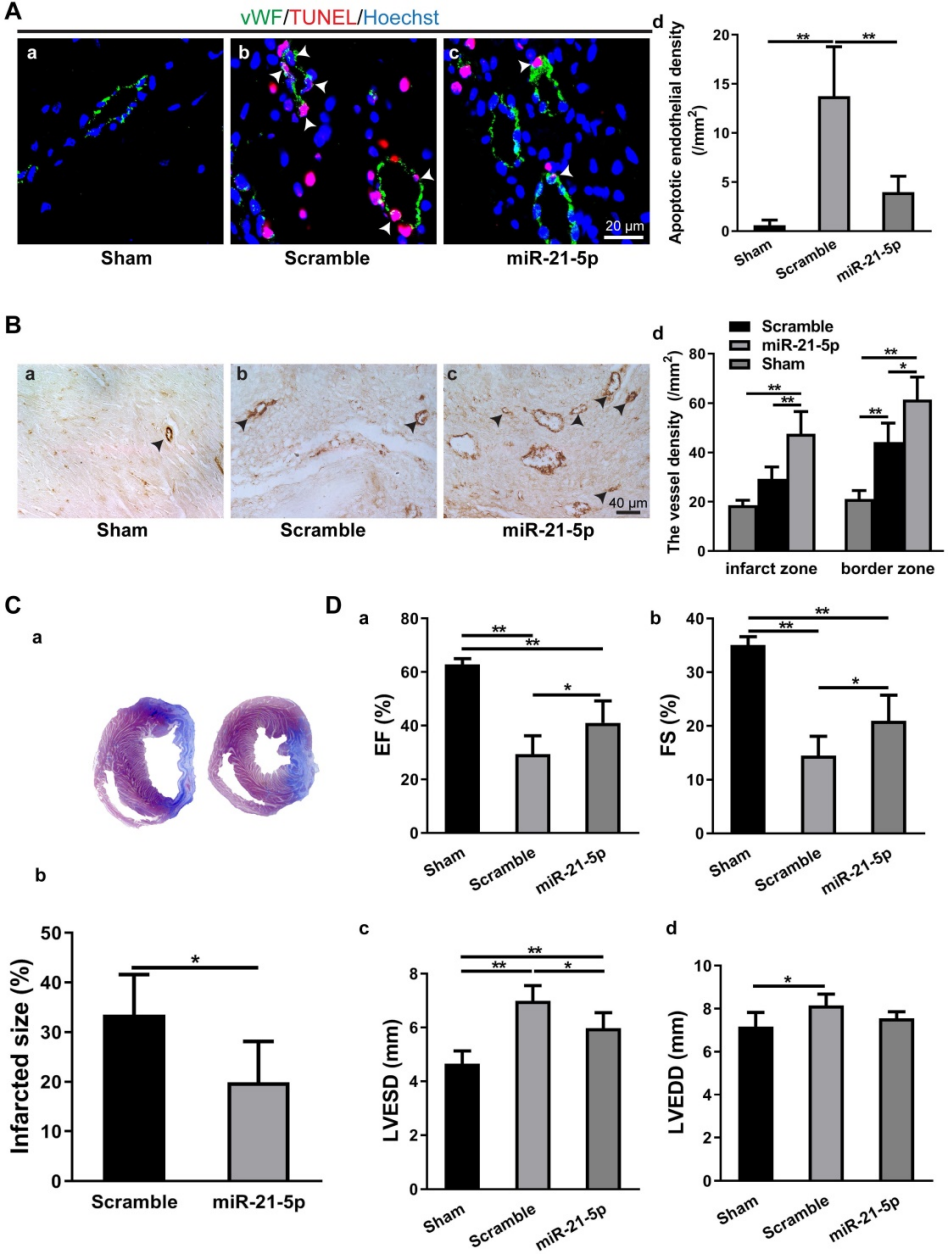
**Intramyocardial injection of miR-21-5p decreased apoptosis in CMECs, increased angiogenesis, reduced infarct size and improved cardiac function. A:** TUNEL staining revealed that the density of apoptotic endothelial cells (vWF^+^) in the miR-21-5p-treated myocardium was significantly lower than that in the scramble control group. n = 5. **B:** Immunohistochemical staining for vWF^+^ showed that blood vessel density in the infarct zone and border zone of the miR-21-5p group was significantly higher than those in the scramble control group. n = 5, 4, and 5 for the sham, scramble and miR-21-5p groups. **C:** The infarct size of the miR-21-5p group was significantly smaller than that of the scramble control group. n = 6 and 6 for the scramble and miR-21-5p groups.** D:** Echocardiography revealed that intramyocardial injection of miR-21-5p significantly increased the (a) ejection fraction and (b) fractional shortening and significantly decreased the final (c) systolic diameter, but not (d) diastolic diameter compared with those in the scramble control group. n = 5, 5, 6 for sham-, scramble- miR-21-5p-group. *: *p* < 0.05, **:* p* < 0.01.

To study the role of miR-21-5p in MI, we transplanted the rno-miR-21-5p agomir into the infarct zone and border zone 30 min after LAD ligation. Seven days later, the parameters for endothelial cell apoptosis, myocardium vessel density, infarct size and cardiac function (EF, FS, LVESD and LVEDD) were analyzed. The TUNEL assay revealed that the density of apoptotic endothelial cells in the miR-21-5p-treated myocardium was significantly lower than that in the scramble miRNA-treated control (Figure 6A). Similarly, the vessel density (vWF^+^) of the miR-21-5p-treated myocardium was significantly higher in the infarct zone and border zone than in that of the scramble miRNA-treated control group (Figure 6B). Consistently, the infarct size was significantly smaller in the miR-21-5p-treated hearts than in the scramble-treated hearts (Figure 6C). In addition, echocardiography for cardiac function in the infarcted hearts revealed that the EF and FS of the miR-21-5p-treated groups were significantly greater than those of the scramble-treated control groups (Figure 6D a&b). Correspondingly, the LVESD, but not the LVEDD of the miR-21-5p-treated groups were significantly smaller than those of the scramble-treated control groups (Figure 6D c&d). These results support the view that the introduction of miR-21-5p into the infarcted myocardium is sufficient to repress apoptosis of endothelial cells, improve cardiac angiogenesis, reduce the infarct size and improve cardiac function in the ischemic myocardium *in vivo*, thus facilitating regeneration following MI.

### miRNA-21-5p-targeted silencing of the *Cdip1* gene downregulated Caspase-3 expression to inhibit CMEC apoptosis in response to ischemic and hypoxic conditions

**Figure 7:**
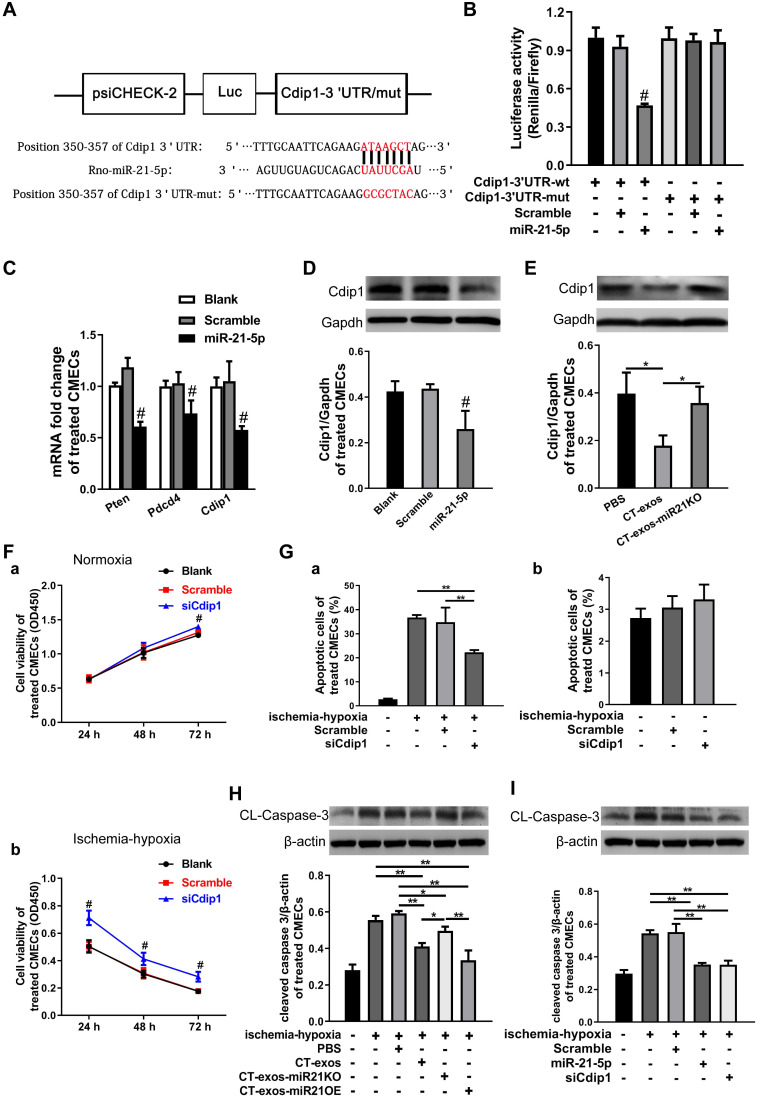
** miRNA-21-5p-targeted silencing of the *Cdip1* gene downregulated Caspase-3 expression to inhibit CMEC apoptosis in response to ischemic and hypoxic conditions. A:** The predicted interaction site and sequences of miRNA-21-5p and the 3' UTR of the *Cdip1* gene.** B:** The dual luciferase reporter assays revealed that the *Cdip1* gene directly interacted with miRNA-21-5p. **C:** The q-PCR results showed that the expression of the selected potential target genes *Cdip1*, *Pten* and *Pdcd4* of miRNA-21-5p was decreased in CMECs after transfection of miRNA-21-5p. n = 3. **D:** Western blots confirmed that overexpression of miR-21-5p in CMECs decreased the protein expression of *Cdip1*. n = 3. **E:** The western blot results revealed that the expression of Cdip1 protein in the CT-exos-miR21KO-treated CMECs was significantly higher than that of the CT-exos-treated CMECs. n = 3. **F:** CCK-8 assays demonstrated that downregulation of Cdip1 in CMECs significantly improved the survival of CMECs under normoxic and ischemic-hypoxic conditions. The study was conducted using two animals in at least three repeated experiments for each individual animal. **G:** Annexin V/PI staining and flow cytometry assays showed that downregulation of Cdip1 in CMECs significantly decreased the apoptosis rate. The study was conducted using two animals in at least three repeated experiments for each individual animal. **H:** The western blot results showed that in an ischemic-hypoxic environment, the expression of the cleaved Caspase-3 (CL-Caspase-3) in CMECs was significantly upregulated in the blank control- and PBS-treated CMECs; however, the CL-Caspase-3 expression level in the CT-exos-treated CMECs and the CT-exos-miR21OE-treated CMECs (upregulation of miR-21-5p) was decreased significantly compared to that of the blank control- and PBS-treated CMECs, while CT-exos-miR21KO treatment for CMECs could reverse the decrease in CL-Caspase-3 expression. In addition, the downregulation induced by CT-exos-miR21OE treatment for CL-Caspase-3 showed an increased trend compared with that of the CT-exos treatment, but the difference was not significant. n = 3. **I:** The western blot results showed that in an ischemic-hypoxic environment, both miRNA-21-5p and siCdip1 treatments in CMECs could significantly downregulate CL-Caspase-3 expression. The effect of downregulated CL-Caspase-3 expression between the miRNA-21-5p and siCdip1 treatments was similar. n = 3. *: *p* < 0.05, **:* p* < 0.01, #: *p* < 0.05 compared with the blank group and scramble group.

The miRNA-21-5p target genes that inhibit CMEC apoptosis were explored. Based on our literature review, *Pdcd4, Pten* and *Faslg* are target genes of miRNA-21-5p in endothelial cells, cardiomyocytes 35, 36 and neurons 37. However, using bioinformatics, we predicted that *Cdip1* was a potential but not yet confirmed novel target gene (Figure 7A), and the seed region of miRNA-21-5p for *Cdip1* is conserved among species (Figure S5C). Therefore, in the present study, we aimed to investigate this novel target gene of miRNA-21-5p and its related novel pathway to inhibit apoptosis of endothelial cells. The 3' UTR of the* Cdip1* gene, which is predicted to interact with miRNA-21-5p, was designed, synthesized, inserted into the psiCHECK-2 reporter vector, and transfected into HEK293T cells. According to the results of our dual luciferase reporter assay, *Cdip1* directly interacted with the miRNA-21-5p mimic (Figure 7B). Furthermore, the expression of the *Cdip1* gene in CMECs that had been transfected with miRNA-21-5p or nonsense miR for 48 h was detected by qPCR and western blot analyses. The expression levels of the *Cdip1* gene were significantly decreased at the mRNA (Figure 7C) and protein levels (Figure 7D). In addition, an assay of the CT-exos- and CT-exos-miR21KO-treated CMECs confirmed that the *Cdip1* protein expression in the CT-exos-miR21KO-treated CMECs was significantly higher than that of the CT-exos-treated CMECs (Figure 7E). Taken together, the findings indicated that *Cdip1* is a novel target gene of miRNA-21-5p and that CT exosomal miRNA-21-5p is able to silence *Cdip1* expression in endothelial cells.

We next examined the biological consequence of knockdown of *Cdip1* expression in CMECs. In an ischemic-hypoxic environment, most CMECs usually react by undergoing apoptosis. It was found that the CCK-8 value of the siCdip1-transfected CMECs in which Cdip1 was knocked down significantly (Fig. S5B) was significantly higher than that of the scramble and blank controls in both normoxic and ischemic-hypoxic conditions (Figure 7F). This result suggested that siCdip1 can improve the survival of CMECs under normoxic and ischemic-hypoxic conditions. Moreover, siCdip1, which was transfected into CMECs, inhibited the apoptotic reaction of CMECs. As a result, the number of apoptotic siCdip1-transfected CMECs was significantly reduced compared with the number of scramble miRNA-transfected CMECs under ischemic-hypoxic conditions (Figure 7G and Figure S4C). Consistently, western blot assays showed that in an ischemic-hypoxic environment, the expression of cleaved Caspase-3 (CL-Caspase3) in CMECs was significantly upregulated in the blank control- and PBS-treated CMECs; however, the CL-Caspase-3 expression level in the CT-exos-treated CMECs and the CT-exos-miR21OE-treated CMECs (upregulation of miR-21-5p) was decreased significantly compared to the blank control and PBS-treated CMECs, while CT-exos-miR21KO treatment of CMECs reversed the decrease in CL-Caspase-3 expression. In addition, the CT-exos-miR21OE treatment-mediated downregulation of CL-Caspase-3 tended to be greater than that of CT-exos treatment; however, the difference was not statistically significant (Figure 7H). Furthermore, in an ischemic-hypoxic environment, western blot analysis confirmed that both miRNA-21-5p and siCdip1 treatment of CMECs significantly downregulated CL-Caspase-3 expression. The effect of downregulated CL-Caspase-3 expression between the miRNA-21-5p and siCdip1 treatments was similar (Figure 7I). Thus, CT exosomal miRNA-21-5p-targeted silencing of the *Cdip1* gene downregulated Caspase-3 expression to inhibit CMEC apoptosis in response to ischemic-hypoxic conditions.

## Discussion

An effective method of promoting survival in endothelial cells and avoiding apoptosis in the ischemic and hypoxic myocardium is a major challenge to the promotion of cardiac angiogenesis in MI regeneration. Our results suggest that cardiac telocytes, novel cardiac interstitial cells, play a potent role in promoting the survival and inhibiting the apoptosis of cardiac endothelial cells (especially CMECs) under MI *via* CT exosomal miRNA-21-5p-targeted *Cdip1* silencing to downregulate caspase-3 activity and facilitate cardiac angiogenesis to improve the regeneration of MI. This inference is supported by the following observations: 1) intramyocardial transplantation of CTs can improve the regeneration of MI by decreasing infarct size and cardiac fibrosis, increasing cardiac angiogenesis and improving cardiac function (present results and previous reports 19, 20); 2) exosome secretion by CTs in the myocardium has been confirmed by TEM and *in vitro* analysis of unique morphological parameters and markers; 3) intramyocardial transplantation of CT exosomes can improve the regeneration of MI by decreasing infarct size and cardiac fibrosis, increasing cardiac angiogenesis and improving cardiac function; 4) cardiac endothelial cells are a target cell of CT exosomes, which has been confirmed by CMEC engulfing assays and transwell assays, and CT exosomes can promote migration and tube formation in CMECs *in vitro*; 5) miRNA-21-5p is the most abundant microRNA in CT exosomes, and various gain- and loss-of-function studies verified that the effects on survival, apoptosis, migration and tube formation in the miRNA-21-5p-deficient exosome-treated CMECs were significantly weaker than those in the CT-exos-treated CMECs. Consistently, the miRNA-21-5p-treated CMECs showed potent survival, inhibition of apoptosis, migration and tube formation compared to the scramble miRNA-treated CMECs under ischemic and hypoxic environments *in vitro*, and miRNA-21-5p treatment alone for MI could significantly decrease apoptotic endothelial cell density, increase vessel density, decrease infarct size and improve cardiac function in the ischemic myocardium *in vivo*; 6) *Cdip1*, a novel target gene of miRNA-21-5p, was shown to have antiapoptotic effects *via* the downregulation of caspase-3 activity, which was confirmed by the following results: miRNA-21-5p paired with *Cdip1* in a luciferase reporter assay, expression of *Cdip1* was silenced at the mRNA and protein levels, the knockdown effect of *Cdip1* expression was lost in miRNA-21-5p-deficient CT exosomes, the antiapoptotic effect was increased upon downregulation of* Cdip1* expression by siCdip1, knockdown of caspase-3 activity using miRNA-21-5p-deficient exosomes reversed the observed effects, and caspase-3 activation was decreased by siCdip1.

Telocytes are a novel type of interstitial cell that have recently been identified in the heart (cardiac telocytes; CTs) 9-20, 25 and other tissues (such as the intestine 38, uterus 39, fallopian tube 40, kidney 41, urinary tract 42, trachea, lung 43, 44, and skin and skeletal muscle 45). Telocytes as well as CTs are identified by their 'hallmark morphology'; under TEM, telocytes are specifically characterized as piriform, spindle-shaped or triangular cells with a nucleus that occupies approximately 25-30% of the cell volume and very long, thin, dichotomously branched protrusions called telopods 21-26. In addition, double immunofluorescence staining and light microscopy showed that CTs are C-kit/CD34-positive cells with very small cell bodies (an approximately 1:1 ratio of the cytoplasm and nucleus) and extremely thin protrusions (a diameter of approximately 1-2 µm) with dilated segments; CTs are easily distinguished from other interstitial cells, such as cardiac fibroblasts 9-20, 25. However, to date, specific markers for the identification of CTs and telocytes in other tissues and organs are still unavailable. In addition, the origin of telocytes during development is still unclear. The lack of a unique molecular marker is one of the important reasons why the developmental origin of telocytes cannot be elucidated. As shown in our recent studies, CTs undergoing cell death in the permanently ischemic myocardium are another unique hallmark for distinguishing CTs from other interstitial cells, such as cardiac fibroblasts 19, 20. Cardiac fibroblasts can survive, proliferate, and differentiate into myofibroblasts without undergoing cell death following an ischemic challenge. The characteristic 'hallmark morphology' and predisposition of CTs to undergo cell death in response to ischemia, along with their expression of molecular markers such as C-kit/CD34, accurately identify CTs present in the myocardium 25. Currently, the functional role of CTs in the physiopathology of the myocardium is still unclear. Limited morphological studies have suggested that CTs might function as niche supporting cells to conduct cell-cell communication among cardiac cells to regulate angiogenesis and inflammation 16, 17. Based on our previous findings that transplantation of CTs following MI decreases the infarct size and cardiac fibrosis and promotes cardiac angiogenesis, we further uncovered a novel mechanism by which exosomes from CTs inhibit the apoptosis and promote the survival of endothelial cells in ischemic and hypoxic environments and improve angiogenesis and regeneration of MI after transplantation of CTs. Simultaneously, the findings of the present study also propose an endogenous role of CTs in regulating the survival and antiapoptotic effects of endothelial cells as well as cardiac angiogenesis *via* CT exosomes and their miRNAs in the pathophysiological myocardium.

Current evidence reveals that the beneficial effects of stem cell therapies are mainly due to paracrine mechanisms instead of direct proliferation and differentiation of transplanted stem cells to cardiac cardiomyocytes 46-49. Accordingly, the results of the present study also support the underlying mechanism of CT therapy for promoting angiogenesis, and the regeneration of MI is mainly attributed to the paracrine actions of CT exosomes. As shown in the present study, exosome secretion occurs and is physiologically necessary in the normal myocardium *in vivo*. The results of the uptake assay and transwell assay clearly demonstrated that CMECs are target cells of CT exosomes. Importantly, the promotion of cardiac angiogenesis, a decrease in infarct size and cardiac fibrosis, and the improvement of cardiac function in MI are achieved concurrently *via* transplantation of CTs, CT exosomes or miRNA-21-5p alone. In fact, exosomes have been well studied as paracrine bioeffectors that conduct cell-cell communication 50. The transplantation of CT exosomes was reported to promote the migration of endothelial cells *in vitro* and angiogenesis in MI 51. However, the study did not identify the biomolecular cargo of CT exosomes that achieved angiogenic effects, and substantial evidence has not been found to support that endothelial cells are the target cell of CT exosomes. In addition, the underlying molecular pathway has not yet been investigated. The significance of the present study is to describe a novel paracrine mechanism by which transplanted CTs achieve enhanced cardiac angiogenesis to facilitate the regeneration of MI, which is mediated by CT exosomal miRNA-21-5p-targeted *Cdip1* silencing to downregulate caspase-3-mediated apoptotic activity. In addition, the present study further revealed that CT exosomes or miRNA-21-5p alone should be an ideal candidate for cell-free therapy to promote angiogenesis *via* the inhibition of apoptosis and the maintenance of survival in endothelial cells in the ischemic myocardium and the facilitation of the regeneration of MI. In addition, the inclusion of highly potent cardiac protective and beneficial miRNAs (such as miRNA-21-5p) allows CTs to be considered an ideal cell type to prepare cardiac protective exosomes by direct culture or further development of gene-modified exosomes. Recently, it was reported that a miRNA-21-enriched exosome prepared by HEK293T cells overexpressing miRNA-21 was able to inhibit apoptosis in cardiomyocytes and endothelial cells by reducing the expression of Pdcd4 and restoring cardiac function in MI 52. For clinical therapy, considering that CTs are a cardiac endogenous cell and that CT exosomes include the highest content of miRNA-21-5p, CTs might be more appropriate as host cells to prepare exosomes enriched with miRNA-21 or other miRNAs using a gene modification strategy compared to a cell line tool.

Exosomes derived from several different cell types, including cardiac progenitor cells 53, cardiosphere-derived cells 54, mouse embryonic stem cells 55, human CD34^+^ stem cells 56, BMMSCs 57, cardiac fibroblasts 58, and human endometrial mesenchymal stem cells 59, have recently been shown to improve myocardial repair. In the present study, we reveal that a novel cardiac interstitial cell type, CTs, plays an important role in facilitating cardiac repair *via* a previously unrecognized CT exosome-dependent paracrine signaling pathway. Thus, the paracrine effects of currently unrecognized interstitial cells, such as CTs, are indispensable for maintaining the integrity of myocardial physiopathology and regenerating the damaged myocardium. Novel strategies used to achieve structural and functional regeneration of the infarcted myocardium should not ignore the parallel survival and regeneration of CTs, which undergo cell death in large numbers and fail to provide their own protective paracrine signaling 19, 20. In addition, recent studies have revealed that transplantation of epicardium-derived cells (EPDCs) into MI preserves cardiac function, attenuates ventricular remodeling, increases cardiac angiogenesis 60 and reinforces cardiac sympathetic innervations in the ischemic zone 61. Considering that during development, EDPCs migrate into the myocardium to differentiate into interstitial cardiac fibroblasts and cardiac mesenchymal cells 62-66, and the preliminary results of our RNA sequencing of CT single cells reveal the interstitial and mesenchymal characteristics of CTs (Figure S1), whether CT is derived from the epicardial lineage is an intriguing question for future study of the telocyte field.

In the present study, CMECs were shown to be recipient cells of CT exosomes, and miRNA-21-5p is a key effector in CT exosomes that inhibits CMEC apoptosis and promotes their survival following CT exosome uptake. CPC-derived exosomes have recently been shown to prevent cardiomyocyte apoptosis through exosomal miR-21 by targeting the *Pdcd4* gene 35. The exosomes derived from human endometrial mesenchymal stem cells exhibited enhanced cardioprotective effects through exosomal miR-21 59. In addition, miR-21-3p, a passenger-strand microRNA, but not the target gene-guiding strand derived from cardiac fibroblast exosomes, triggered cardiomyocyte hypertrophy by silencing the sorbin and SH3 domain-containing protein 2 (*Sorbs2*) and PDZ and LIM domain 5 (*Pdlim5*) genes 67. Based on our results and the results reported by other groups, miR-21-5p is derived from multiple cell sources in the myocardium, CTs and cardiac progenitor cells and functions as an important cardioprotective effector to facilitate the repair of the infarcted myocardium. The finding that miR-21-3p, but not miR-21-5p, is present in cardiac fibroblast exosomes at high levels also supports the hypothesis that CTs are unique interstitial cells distinct from cardiac fibroblasts. The most abundant miRNA cargo of CT-derived exosomes was miR-21-5p, whereas miR-21-3p was not detected in CT-derived exosomes. Thus, unlike cardiac fibroblasts, CTs play a unique role in regulating cardiac cell communication and physiopathology *via* exosomal miR-21-5p. Importantly, the present study is the first to reveal a novel mechanism by which exosomal miR-21-5p derived from CTs inhibits CMEC apoptosis *via* the targeted silencing of the *Cdip1* gene under hypoxic and ischemic conditions, and transfection of miR-21-5p decreases the infarct size, improves cardiac function, increases cardiac angiogenesis and improves MI regeneration. In addition, miR-21 alone was recently reported to promote proliferation and tube formation by suppressing PTEN expression and enhancing VEGF expression 68. Based on our and other results, miR-21-5p is a potent cardioprotective effector for the inhibition of apoptosis in CMECs and the promotion of angiogenesis that facilitates the regeneration of MI, and its therapeutic effects should be studied more extensively.

*Cdip1* was identified as a p53 target gene that is upregulated upon DNA damage and is a key downstream effector of p53-dependent apoptosis. The expression of Cdip1 alone is sufficient to induce apoptosis. Cdip1-dependent apoptosis is accompanied by caspase-8 cleavage, identifying the extrinsic cell death pathway as a mediator of the Cdip1-induced death signal. In addition, TNF-α expression is tightly correlated with Cdip1 expression, and the inhibition of TNF-α signaling attenuates Cdip1-dependent apoptosis. Moreover, TNF-α is upregulated in response to p53 and p53-induced genotoxic stress in a Cdip1-dependent manner. Thus, Cdip1 provides a link between p53-mediated intrinsic and death receptor-mediated extrinsic apoptotic signaling to induce apoptosis 69. Cdip1 was recently shown to act as a key transducer of endoplasmic reticulum (ER) stress signals through its interaction with B-cell receptor-associated protein 31 (BAP31) at the ER membrane. Upon ER stress, Cdip1 expression is induced, increasing its association with BAP31 at the ER membrane. The Cdip1-Bap31 complex is critical for ER stress-mediated cell death and for the formation of the BAP31-Bcl-2 complex. The recruitment of Bcl-2 to the Bap31-Cdip1 complex, as well as Cdip1-dependent truncated Bid (tBid) and caspase-8 activation, contributes to BAX oligomerization, and genetic knockout of Cdip1 in mice led to impaired responses to ER stress-mediated apoptosis. Thus, the Cdip1/BAP31-mediated regulation of the mitochondrial apoptotic pathway represents a mechanism for establishing ER-mitochondrial crosstalk during ER stress-mediated apoptosis signaling 70. Recently, it was reported that miR-133a-3p suppresses cardiomyocyte apoptosis by targeting *Cdip1* 71. In the present study, we further demonstrated that miRNA-21-5p targeted *Cdip1* silencing to downregulate caspase-3-mediated apoptotic activity in endothelial cells. Our findings, combined with the aforementioned functions of Cdip1, explain the underlying downstream molecular mechanism by which miR-21-5p-targeted silencing of the *Cdip1* gene in CMECs decreases apoptosis and increases survival under hypoxic and ischemic conditions. This finding suggests that exosomal miR-21-5p from CTs is a good *in vivo* regulator that decreases the expression of the *Cdip1* gene and the activity of caspase-3, a key apoptotic player, and silencing of the *Cdip1* gene *via* exosomal miR-21-5p may be a potential strategy to improve the proapoptotic microenvironment of the ischemic myocardium and subsequently to facilitate cardiac angiogenesis and regeneration following MI by promoting the survival of transplanted cells and resident cells (such as CMECs). In fact, we also found that CT exosomes can be engulfed in cardiomyocytes and cardiac fibroblasts (data not shown). Importantly, as the sequence of miRNA-21-5p and the seed domain of miRNA-21-5p for *Cdip1* are conserved among species, the findings and the underlying molecular mechanism of this study are applicable for humans.

## Conclusions

In summary, the present study is the first to show that CTs exert strong therapeutic effects on MI *via* the potent paracrine effects of CT exosomes to inhibit apoptosis and promote survival in endothelial cells, especially CMECs, to achieve cardiac angiogenesis. Moreover, we revealed a novel cellular and molecular mechanism of CTs that underlies CT-endothelial cell communication to inhibit apoptosis and promote survival in endothelial cells in the pathophysiological myocardium *via* CT exosomes and their miRNA cargo. Thus, CT exosomal miRNA-21-5p targeted and silenced the* Cdip1* gene and thus down-regulated the activated caspase-3, which then inhibited the apoptosis of recipient endothelial cells under ischemic and hypoxic conditions, facilitating angiogenesis and regeneration following MI. We believe that these novel findings and the discovery of the cellular and molecular mechanisms will provide new opportunities to tailor novel cardiac cell therapies and cell-free therapies for the functional and structural regeneration of the injured myocardium.

## Supplementary Material

Supplementary figures and tables.

Supplementary vedios.

## Abbreviations

AFM: atomic force microscopy
CAIZ: collagen area of the infarct zone
Cdip1: cell death inducing p53 target 1
CMECs: cardiac microvascular endothelial cells
Cl-Caspase3: cleaved Caspase3
CTs: cardiac telocytes
CT-exos: CT-derived exosomes
CT-exos-miR21KO: miR-21-5p-deficient CT exosomes
CT-exos-miR21OE: miR-21-5p-overexpressing CT exosomes
CTs-miR21KO: CTs transfected with miR-21-5p antagomir
EF: ejection fraction
ER: endoplasmic reticulum
Faslg: Fas ligand
FS: fractional shortening
GAPDH: glyceraldehyde-3-phosphate dehydrogenase
HF: heart failure
LAD: left anterior descending coronary artery
LVEDD: left ventricular end-diastolic diameter
LVESD: left ventricular end-systolic diameter
MI: myocardial infarction
PBS: phosphate-buffered saline
Pdcd4: programmed cell death 4
Pten: phosphatase and tensin homolog
PVCA: perivascular collagen volume area of the noninfarct zone
q-PCR: real-time quantitative-PCR
SEM: scanning electron microscopy
TEM: transmission electron microscopy
3'UTRs: 3' untranslated regions
