# Supplementary material for: Cardiac telocytes inhibit cardiac microvascular endothelial cell apoptosis through exosomal miRNA-21-5p-targeted cdip1 silencing to improve angiogenesis following myocardial infarction
Source: Theranostics. 2021 Jan 1;11(1):268–91. doi: 10.7150/thno.47021 (PMC7681094; doi:10.7150/thno.47021)
Supplement: Supplementary file 1 — Supplementary figures and tables. [file thnov11p0268s1.pdf]

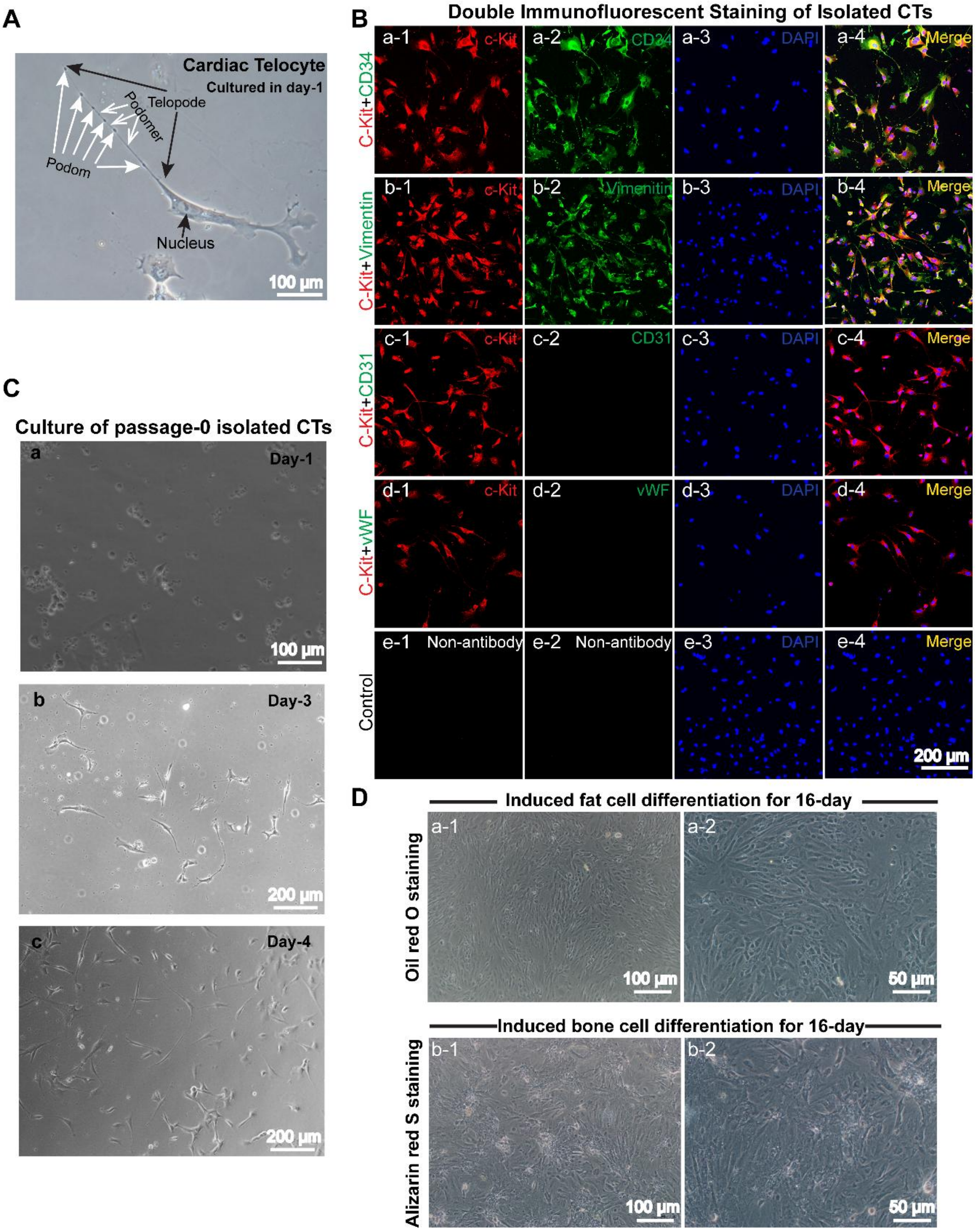

E

Gene name	Single CT (Expression level of FPKM)										
	CT1	CT2	CT3	CT4	CT5	CT6	CT7	CT8	CT9	CT10	CT11
Oct-4	ND	ND	ND	ND	ND	ND	ND	ND	ND	ND	ND
Ssea-1	ND	ND	ND	ND	ND	ND	ND	ND	ND	ND	ND
Sca-1	ND	ND	ND	ND	ND	ND	ND	ND	ND	ND	ND
Cd105	120.5	114.1	62.2	34.2	69.1	76.5	ND	83.2	101.5	66.0	30.2
Cd73	94.9	17.9	23.1	24.4	29.1	12.5	ND	43.7	54.1	30.4	ND
Col1a1	1908.5	2444.7	1917.6	1261.1	1369.1	1273.0	18.6	968.0	970.9	1089.8	1402.9

ND: Nondetectable （FPKM ≤ 10）

Figure S1. Phenotypic confirmation of CTs. A: The representative morphology of the earliest passage (Passage-0) of cultured CTs. The cells had piriform/spindle/triangular cell bodies containing long and slender telopods with the alternation of thick segments (podoms) and thin segments (podomers) that match the unique morphology of telocytes. **B:** Double immunofluorescence staining (anti-c-Kit+anti-CD34; anti-c-Kit+anti-vimentin; anti-c-Kit+anti-CD31; anti-c-Kit+anti-vWF) showed that the collected cells were positive for c-Kit, CD34 and vimentin, while they were negative for the endothelial cell markers CD31 and vWF. **C:** The cultured CTs did not show colony formation. The cultured CTs entered growth arrest at approximately 10-15 passages when they were subcultured serially (data not shown here). **D:** Negative Oil Red O staining of the collected CTs (passages 1~2), which underwent fat cell differentiation for 16 days (a1, 2). Negative Alizarin red S staining of the collected CTs (passages 1~2), which underwent bone cell differentiation for 16 days (b1, 2). The results showed that CTs failed to be induced to differentiate into mesoderm-derived cells, such as bone and fat cells. **E:** Single-cell RNA sequencing for 11 CTs revealed that all 11 collected single cells were negative for the common stem cell markers Oct-4, SSEA-1 and Sca-1. In addition, the cells were positive for CD105, CD73 and Col1a1 (Figure S1E), which are commonly expressed by mesenchymal linage-derived interstitial cells. The positive expression of single-cell sequencing was set at ≥ 10 FPKM. All of the above evidence strongly supports that the CTs that were applied in this study are consistent with the commonly accepted morphology and molecular marker features of telocytes. These cells were more similar to interstitial cells than endothelial cells and progenitor stem cells.

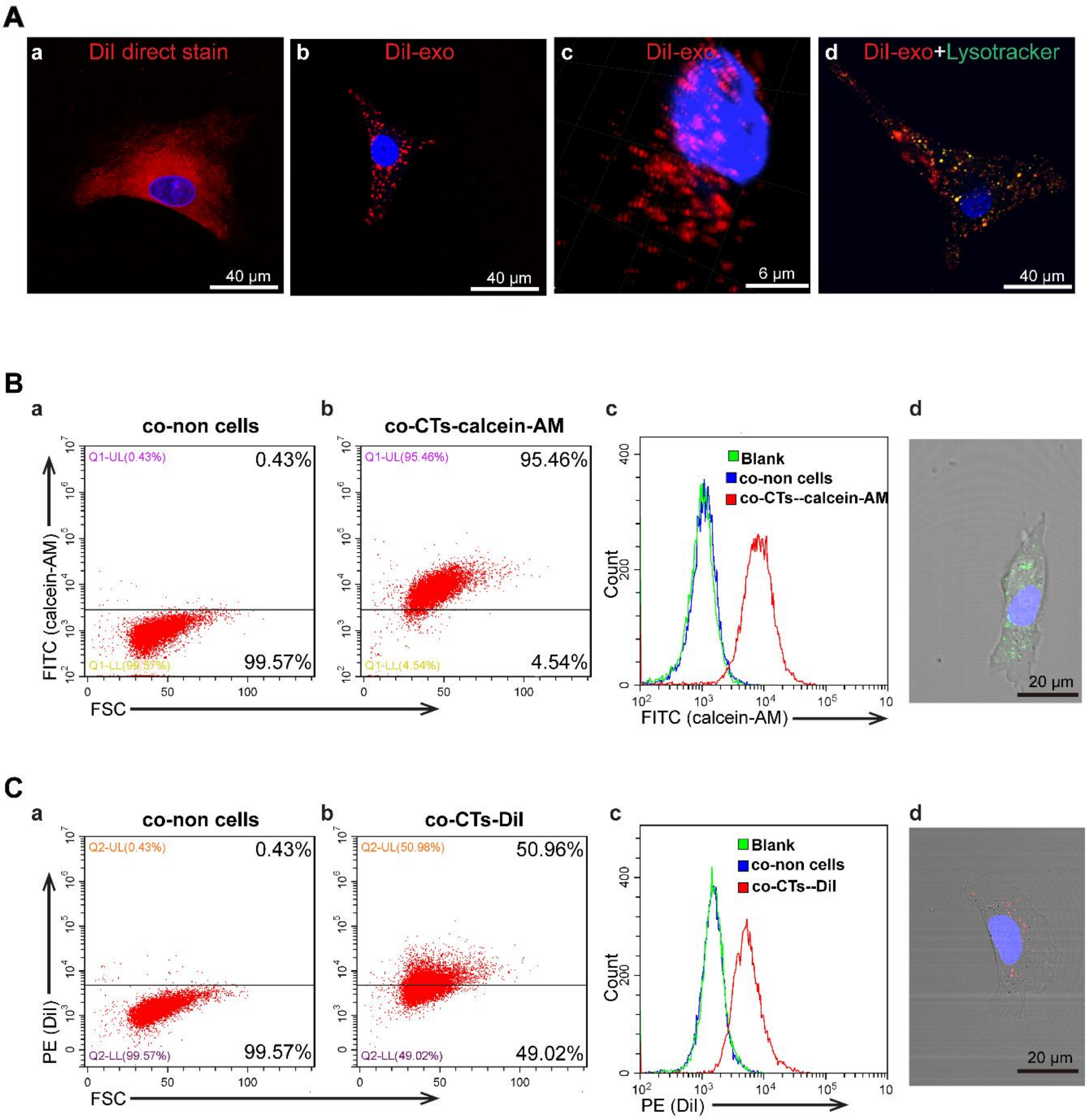

Figure S2. Uptake assay, LysoTracker staining and transwell assay for exosomes secreted by CTs. A: (a) DiI-labeled CMECs. (b) CMECs take up DiI-labeled CT-exos (6 h). (c) A 3D reconstruction of the images shown in b. (d) CMECs were allowed to take up DiI-labeled CT-exos for 6 h, and then, the cells were incubated with LysoTracker (lysosome probe; green) for 2 h. Most of the CT-exosomes were not degraded by lysosomes 8 h after uptake. **B-C:** The transwell assay contained CTs with good washing after they were stained with calcein (green) (B) and Dil (Red) (C) in the upper chamber, and the cells were cocultured with lower chamber, which was seeded with

CMCEs, for 48 h. Flow cytometry revealed that the green- and red-positive CMCEs in the lower chamber comprised approximately 95% and 51%, respectively. In addition, green- or red-positive CMCEs were only found in the calcein straining-CT-treated group or the Dil staining-CT-treated group, respectively (B a-c; C a-c). In parallel, scattered green and red fluorescent dots were found in the treated CMCEs of the lower chamber by microscopy (B d; C d). The results document that CT-derived exosomes can be secreted, targeted and engulfed by CMECs.

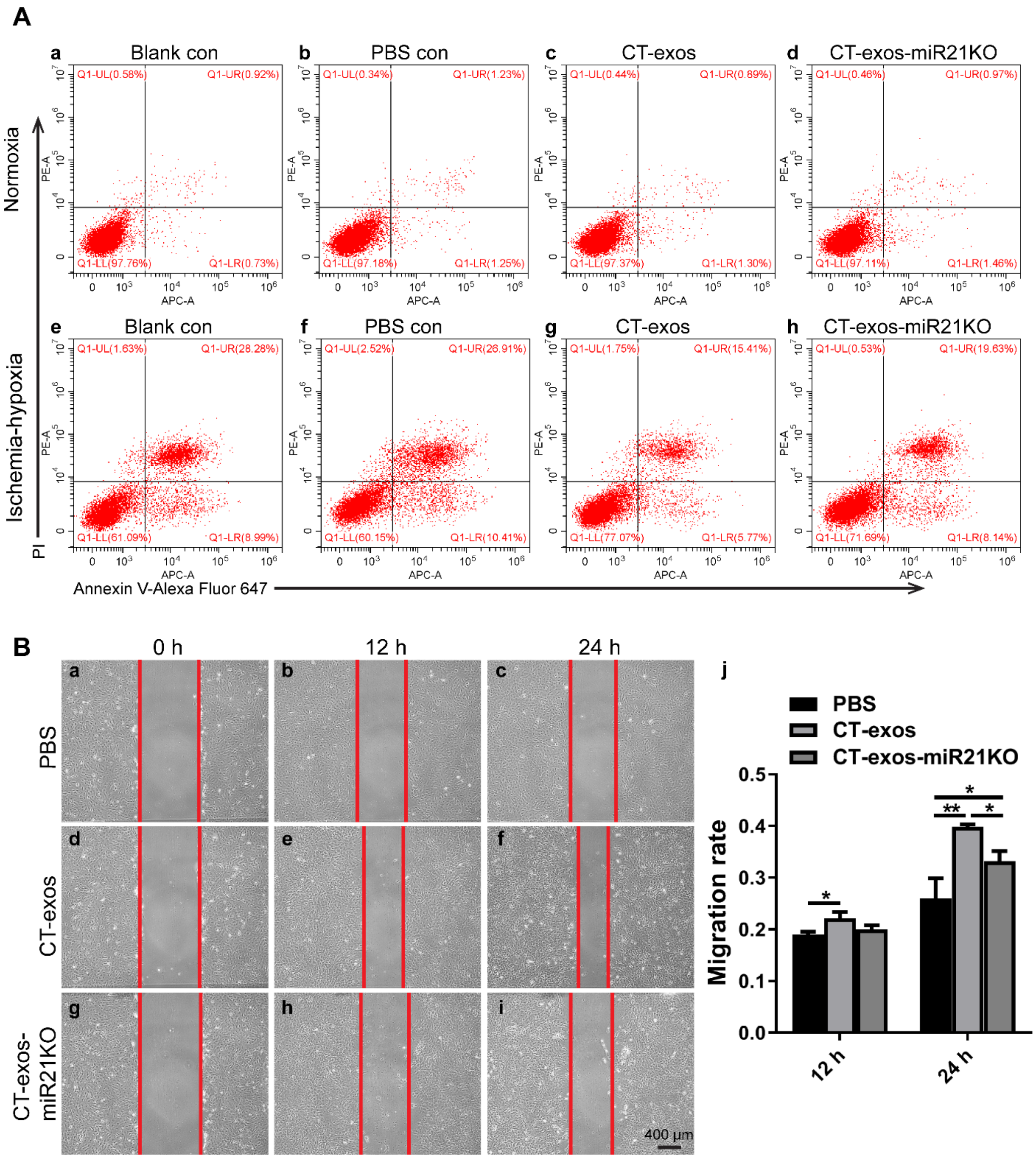

Figure S3. Flow cytometry apoptotic assay and migration assay for CT-exos-treated CMECs. **A:** Representative flow cytometry analysis figures of Annexin V/PI staining for the CT-exos-, CT-exos-miR21KO-, PBS- or blank-treated CMECs cultured under normoxic and ischemic-hypoxic conditions for 24 h (semiquantitative results are shown in Figure 4B). The study was conducted using two animals in at least three repeat experiments for each individual animal. **B:**

The wound healing migration assay of CMECs incubated with CT-exos, CT-exos-miR21KO or PBS. n = 3. *: $p<0.05$. **: $p<0.01$.

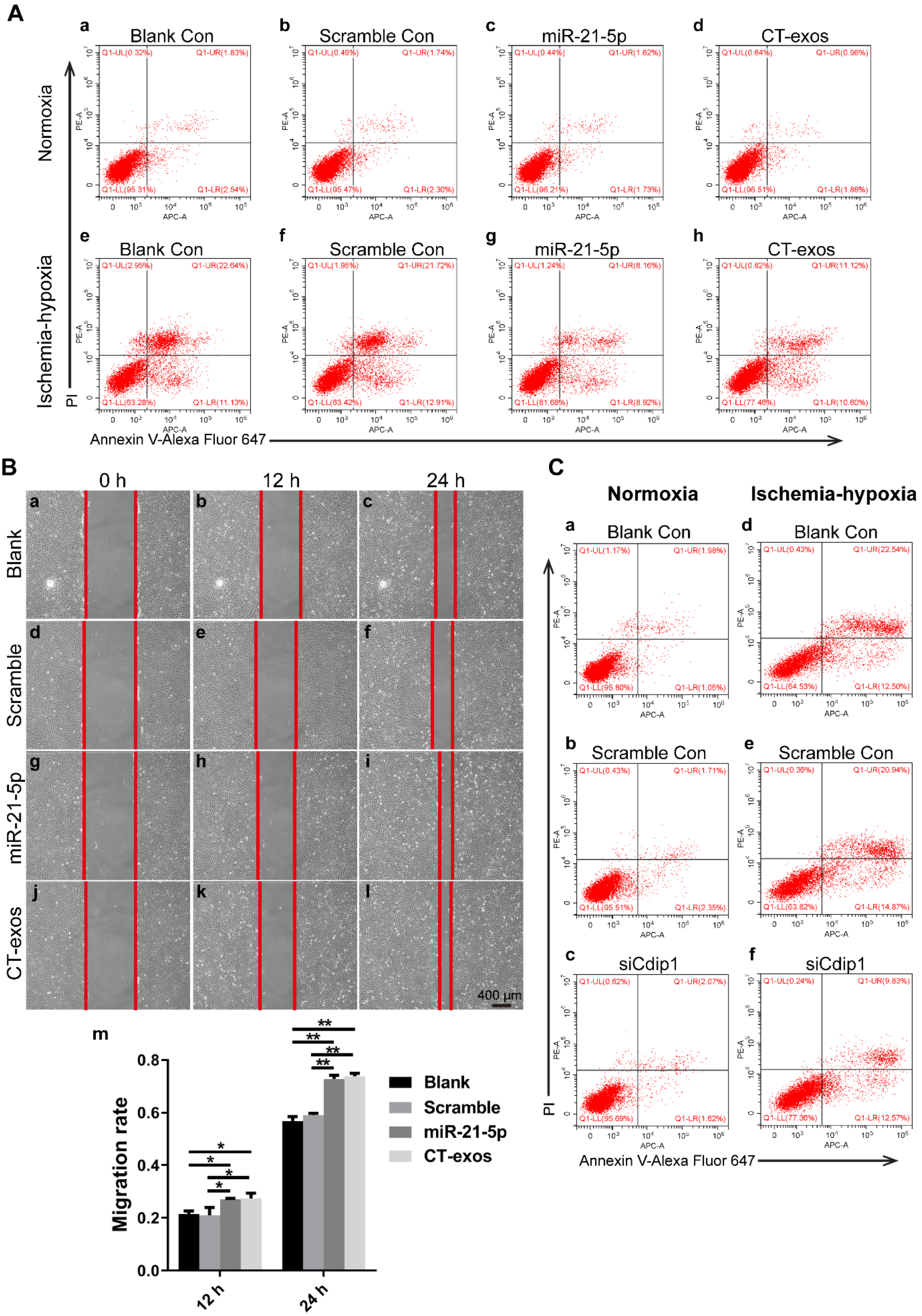

A
Normoxia
Ischemia-hypoxia
a Blank Con
b Scramble Con
c miR-21-5p
d CT-exos
e Blank Con
f Scramble Con
g miR-21-5p
h CT-exos
PI
Annexin V-Alexa Fluor 647
PE-A
APC-A
B
0 h
12 h
24 h
Blank
Scramble
miR-21-5p
CT-exos
400 μm
m
Migration rate
12 h
24 h
Blank
Scramble
miR-21-5p
CT-exos
C
Normoxia
Ischemia-hypoxia
a Blank Con
d Blank Con
b Scramble Con
e Scramble Con
c siCdip1
f siCdip1
PI
Annexin V-Alexa Fluor 647

Figure S4. Flow cytometry apoptotic assay and migration assay for miR-21-5p-treated CMECs. A: Representative flow cytometry analysis figures of Annexin V/PI staining for the miR-21-5p-, scramble-, CT-exos-treated- or blank-CMECs cultured under normoxic and ischemic-hypoxic conditions for 24 h (semiquantitative results are shown in Figure 5B). **B:** The wound healing migration assays of CMECs incubated with miR-21-5p, scramble, CT-exos or blank. *: p<0.05. **: p<0.01. The study was conducted using two animals in at least three repeat experiments observed for each individual animal. **C:** Representative flow cytometry analysis figures of Annexin V/PI staining for the siCdip1-, scramble- or blank-treated CMECs cultured under normoxic and ischemic-hypoxic conditions for 24 h (semiquantitative results are shown in Figure 7G). The study was conducted using two animals in at least three repeat experiments observed for each individual animal.

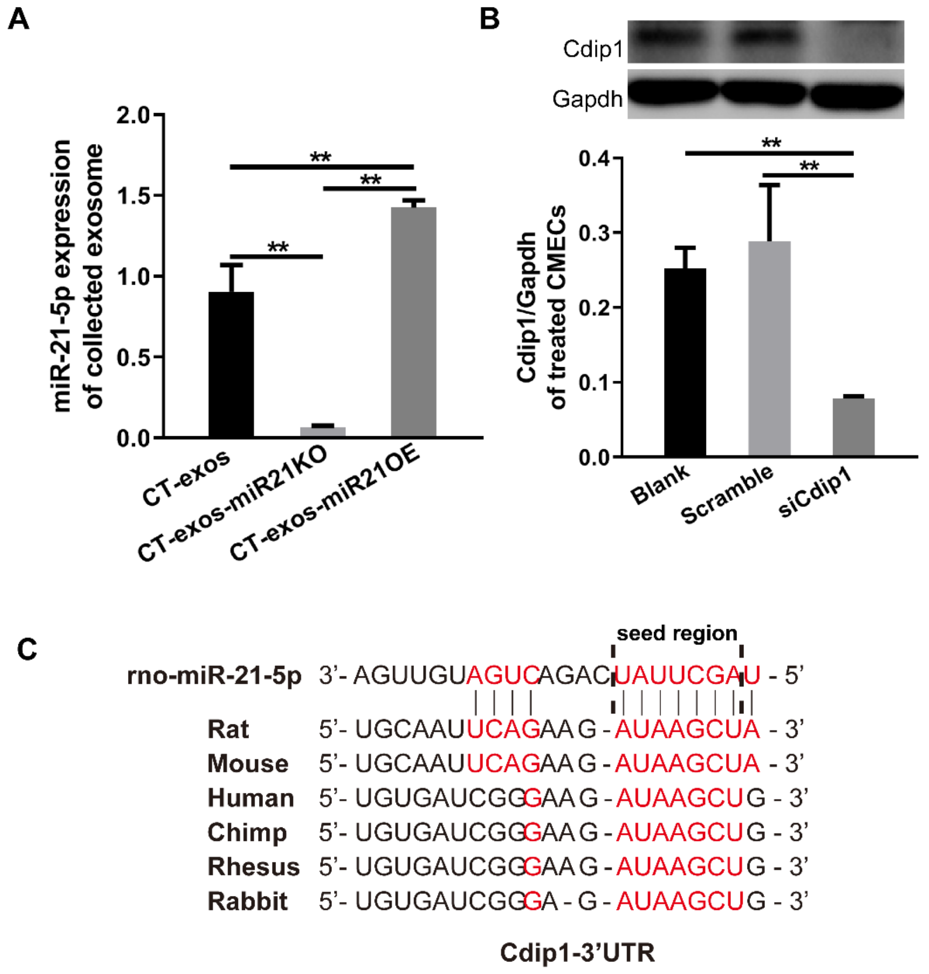

Figure S5. Comparison of miR-21-5p expression in the CT-exos, CT-exos-miR21KO and CT-exos-miR21OE groups, analysis of the knockdown efficiency of siCdip1, and homology analysis of the miR-21-5p targeting site in the CDIP1 3' UTR. A: The miR-21-5p level of the CT-exos-miR21KO-exo group was significantly lower than that of the CT-exos group, while the miR-21-5p level of the CT-exos-miR21OE group was significantly higher than that of the CT-exos group. **B:** The expression of Cdip1 was significantly knocked down in the siCdip1-treated CMECs. **C:** The seed region of miRNA-21-5p for Cdip1 is conserved among species. **: p<0.01.
